# Roles of inflammation and metabolic dysfunction in cardiovascular diseases: molecular mechanisms and therapeutic targets

**DOI:** 10.1186/s43556-026-00599-x

**Published:** 2026-09-24

**Authors:** Yue Pan, Wan-Hong Li, Wei Zhang, Wan-Jing Ma, Hui Sun

**Affiliations:** 1 Experiment Teaching Center, College of Pharmacy, Harbin Medical University, Harbin, 150081 China; 2 State Key Laboratory of Bioactive Substance and Function of Natural Medicines, Institute of Materia Medica, Chinese Academy of Medical Sciences & Peking Union Medical College, Beijing, China

**Keywords:** Metabolism–inflammation crosstalk, Cardiovascular disease, Metabolic reprogramming, Cardiovascular inflammation, Therapeutic targets, Amino acid metabolism, Glucose metabolism, Fatty acid metabolism

## Abstract

Cardiovascular disease progression is shaped by a close interaction between metabolic adaptation and inflammatory signaling. In injured cardiac and vascular tissues, ischemia, pressure overload, lipid stress and metabolic excess reshape how cardiomyocytes, endothelial cells, immune cells and fibroblasts sense damage and communicate with their microenvironment. Cardiovascular inflammation follows a staged tissue response, from danger-signal sensing and nuclear factor-κB-related priming to inflammasome activation, cytokine amplification, leukocyte recruitment, efferocytosis and chronic remodeling. Metabolic reprogramming shapes this sequence by altering substrate use, redox control, biosynthetic routing and metabolite signaling. A major challenge is that glycolysis, fatty acid oxidation and amino-acid metabolism do not carry fixed biological meanings; their effects depend on the cell type, measurement layer and disease phase in which they occur. This review examines metabolism–inflammation crosstalk in cardiovascular disease by linking core inflammatory pathways with metabolic sensors, immune-cell metabolic reprogramming and signaling metabolites, then comparing disease-specific mechanisms in myocardial infarction/ischemia–reperfusion injury, heart failure, atherosclerosis, hypertrophic remodeling and myocardial fibrosis. We emphasize how apparently similar metabolic shifts can represent adaptive repair, inflammatory amplification or maladaptive remodeling in different compartments. The therapeutic discussion separates pathway activity from targetability and uses anti-inflammatory trials and metabolic interventions to define when patient selection, pathway engagement and safety considerations support targeted treatment.

## Introduction

Research on cardiovascular metabolism has expanded from a primary focus on ATP generation, substrate selection and energy efficiency to cover a broader range of topics now, such as how metabolic processes regulate cell signaling, redox homeostasis and inflammation [1–4]. Traditionally, changes in glucose and fatty acid utilization have been regarded primarily as adaptations to altered energetic demands and substrate availability in the diseased heart [1, 2]. Recently, research in immunometabolism has shown that metabolic pathways and their intermediates can also regulate the activation of immune cells and inflammatory signals [3, 4]. An extended view of cardiovascular injury is needed because ischemia, pressure overload, lipid stress, metabolic disorders and other factors change substrate utilization and alter the perception and communication of damage among cardiomyocytes, endothelial cells, immune cells and fibroblasts. Therefore, only knowing that glycolysis, fatty acid oxidation (FAO) or amino acid metabolism is active is insufficient to determine its biological significance; rather, it needs to be understood in the context of the cellular environment and the stage of injury and subsequent tissue response [1–4].

The inflammatory component of this interaction has a recognizable temporal organization. Danger-signal sensing and nuclear factor-κB (NF-κB)-related priming initiate inflammatory response; the inflammasome and cytokine pathways amplify the response; leukocyte recruitment, clearance of damaged cells and efferocytosis subsequently affect whether inflammation resolves, supports repair or progresses to persistent remodeling [5–9]. Metabolic remodeling can change the extent, duration and cellular consequences of all these stages. A metabolic switch that promotes survival in acute injury may be detrimental if it continues in a different stage of the disease, occurs in a different cellular context, or produces metabolites that sustain inflammatory signals. Therefore, the current evidence supports the view that metabolic reprogramming is a stress-responsive modification of substrate utilization, redox regulation, biosynthetic routing and metabolite signaling, rather than a simple switch in fuel sources [1–4].

Recently, a large number of studies have been conducted on the connection between metabolic reprogramming and specific inflammatory and structural changes in major cardiovascular diseases, but the mechanistic significance of these changes remains highly context dependent. Glycolytic remodeling can support the adaptation and survival of cardiomyocytes in myocardial infarction and ischemia–reperfusion injury (MI/IRI), and changes in lipid and sphingolipid metabolism may also occur during the inflammatory response after injury [10–12]. In atherosclerosis (AS), enhanced glycolytic programs in vascular and immune cells are more closely associated with the progression of inflammatory plaques than with a general increase in glucose utilization [13]. In hypertrophic and fibrotic remodeling, patient hypertrophic cardiomyopathy (HCM) data and pressure-overload models [14–17], as well as fibroblast extracellular matrix (ECM) programs [18, 19], should be handled as related but non-equivalent evidence streams. Together, these differences create an important challenge for synthesis: changes in enzyme expression, metabolite abundance, metabolic flux and cell-state-specific metabolic programs are not interchangeable indicators of mechanism, and their interpretations may vary among different experimental systems and disease stages [20, 21]. Therefore, this review will examine metabolic observations in the context of specific cell populations, disease stages, metabolic indicators and measurable tissue responses to evaluate mechanistic claims.

Therapeutic interpretation follows the same evidentiary logic. Circulating metabolites, altered enzyme levels or inflammatory markers may be indicative of a disease-associated state, but they do not establish causality, tissue-specific activity or therapeutic tractability. By combining the temporal stages of cardiovascular inflammation with cellular context, disease setting and strength of metabolic evidence, this review aims to differentiate between adaptive metabolic responses and mechanisms that directly cause inflammatory injury and remodeling. First, the review introduces the stages of the inflammatory response in cardiovascular disease, then offers guidelines for interpreting metabolic evidence, explores the common molecular mechanisms linking metabolism and inflammation, compares disease-specific patterns of metabolic-inflammatory crosstalk, and finally evaluates therapeutic strategies and the evidence supporting their translation.

## Core cardiovascular inflammatory pathways: from danger-signal sensing to chronic remodeling

Cardiovascular inflammation is organized here as a staged tissue response, not a list of isolated cytokines. Danger signals first prime inflammatory transcription, inflammasome and cytokine modules then amplify the response, and recruited immune cells decide whether inflammation resolves or becomes chronic remodeling [22, 23]. This order keeps NF-κB, Toll-like receptor 4 (TLR4), NOD-like receptor family pyrin domain-containing 3 (NLRP3), interleukin-1β (IL-1β), interleukin-6 (IL-6), tumor necrosis factor-α (TNF-α), interleukin-10 (IL-10), chemokines, adhesion molecules, macrophages and T cells in their biological positions instead of collapsing them into interchangeable inflammatory labels [5, 6]. It also prepares the metabolic argument developed below: the same inflammatory pathway can prime defense, amplify injury or support repair according to cell type, timing and tissue context.

### Danger-signal sensing and inflammatory priming

Inflammatory priming begins when tissue stress is converted into signals that immune and vascular cells can read. In myocardial ischemia–reperfusion injury (IRI), damaged cardiomyocytes release high-mobility group box 1 (HMGB1), heat shock proteins (HSPs), extracellular adenosine triphosphate (ATP), nuclear DNA, mitochondrial DNA (mtDNA) and RNA, which act as damage-associated molecular patterns (DAMPs) and activate pattern-recognition receptors (PRRs), leading to NF-κB-dependent cytokine release [24]. Vascular disease follows a related but more chronic logic: matrix fragments, intracellular proteins, nucleic acids and other endogenous danger molecules can activate Toll-like receptors (TLRs), receptor for advanced glycation end products (RAGE), cyclic GMP-AMP synthase-stimulator of interferon genes (cGAS-STING) or NLRP3-related pathways and thereby connect cardiovascular risk factors to sterile vascular inflammation [25].

TLR4 is the most practical example of this priming layer. In myocardial inflammation, TLR4 engagement activates myeloid differentiation primary response 88 (MyD88)-dependent signaling through interleukin-1 receptor-associated kinase (IRAK), TNF receptor-associated factor 6 (TRAF6), transforming growth factor-β-activated kinase 1 (TAK1) and the IκB kinase (IKK) complex, resulting in NF-κB and activator protein 1 (AP-1) activation and induction of TNF, IL-1, IL-6, chemokines and cell-surface molecules [26]. Its therapeutic implication changes with the disease setting, because TIR-domain-containing adapter-inducing interferon-β (TRIF)/interferon regulatory factor 3 (IRF3)–interferon-β (IFN-β) signaling may support antiviral defense in viral myocarditis. Yang et al. also note that TLR4 can signal through TRIF/IRF3 and IFN-β, especially in viral myocarditis, so complete TLR4 blockade may weaken host defense as well as reduce inflammation [26].

Endothelial activation is the vascular expression of this priming process. Risk factors and inflammatory stress reduce nitric oxide (NO) bioavailability, increase endothelial permeability and allow lipoprotein retention in the subendothelial space [5]. Activated endothelium expresses vascular cell adhesion molecule-1 (VCAM-1), intercellular adhesion molecule-1 (ICAM-1) and selectins, while chemokines guide leukocyte rolling, firm adhesion and trans-endothelial migration [8, 27]. Reactive oxygen species (ROS) are relevant at this early stage because oxidative stress can contribute to endothelial dysfunction and later provide one route toward NLRP3 inflammasome activation [5, 28].

### Inflammasome and cytokine amplification

The inflammasome layer begins after priming. NLRP3 forms a complex with apoptosis-associated speck-like protein containing a CARD (ASC) and procaspase-1; after activation, caspase-1 cleaves pro-IL-1β and pro-IL-18 and promotes gasdermin D (GSDMD)-dependent pyroptosis [28]. This two-step structure matters. NF-κB-related priming increases NLRP3, pro-IL-1β and pro-interleukin-18 (pro-IL-18), whereas the second signal may come from K^+^ efflux, Ca2^+^ signaling, ROS, organelle damage, extracellular ATP, cholesterol crystals or intracellular DAMPs [6, 28]. NLRP3 becomes mechanistically specific only when linked to the initiating danger signal instead of being used as a synonym for inflammation.

IL-1β links this module to broader cytokine amplification. In atherosclerosis (AS), NF-κB-primed macrophages exposed to cholesterol crystals or cellular hypoxia can activate NLRP3, generate IL-1β and IL-18, and promote IL-6 production; IL-6 then drives hepatic C-reactive protein (CRP) generation and expands the inflammatory signal beyond the plaque [6]. Abbate et al. place IL-1 even higher in the innate immune hierarchy: IL-1α and IL-1β signal through interleukin-1 receptor type 1 (IL-1R1)/IL-1 receptor accessory protein and MyD88 to activate NF-κB, while interleukin-1 receptor antagonist and soluble receptors counterbalance this pathway [7]. This explains why IL-1 inhibition has therapeutic relevance, but it also warns against using circulating IL-1β alone as a surrogate for tissue activity [7].

TNF-α, IL-6 and IL-10 fit this network only when their roles are separated. In heart failure (HF), TNF-α, IL-1, IL-6 and CRP are increased and relate to disease severity and prognosis; the inflammasome may amplify this response through TNF-α and inducible nitric oxide synthase, linking low-grade inflammation to remodeling [29, 30]. IL-10 occupies a different position. In post-MI mice, IL-10 infusion reduced left ventricular (LV) dilation, improved ejection fraction, decreased infarct macrophage numbers and shifted macrophage/fibroblast behavior toward wound repair, but these data define experimental repair biology and do not establish a general IL-10 therapy for cardiovascular disease (CVD) [31].

### Leukocyte recruitment, immune-cell states and resolution

Once priming and cytokine amplification have started, inflammation becomes a cellular process. Chemokines create gradients for immune-cell recruitment; in AS, CCL2/CCR2, CXCL1/CXCR2, CCL5, CXCL10 and CX3CL1/CX3CR1 illustrate how leukocytes are attracted, retained or positioned within lesions [8]. Their role is wider than chemotaxis: some axes influence foam-cell formation, adaptive immune-cell homing and fibrous-cap stability, which is why chemokine blockade needs cell- and stage-specific interpretation [8]. Adhesion molecules occupy the same transition zone: VCAM-1 and ICAM-1 translate endothelial activation into leukocyte adhesion and transmigration [27].

The recruited cells should not be reduced to a fixed M1/M2 scheme. Human macrophage and plaque studies further show that iron-handling programs, M1/M2-associated responses and inflammatory profiles vary with activation context, tissue and sex [32–34]. Healthy and diseased hearts contain heterogeneous innate and adaptive immune cells; resident cardiac macrophages participate in homeostasis, electrical coupling and cardiomyocyte maintenance, whereas recruited monocytes and macrophages dominate many injury responses [35–39]. After MI, neutrophils and monocytes enter the myocardium in a staged manner, supporting debris removal, cytokine production, angiogenesis and scar maturation when the response is proportionate, but driving adverse remodeling when it persists [40–43]. Single-cell profiling further shows that post-MI neutrophils are temporally heterogeneous rather than a uniform population [44]. T cells add a slower layer, particularly in chronic plaque inflammation and post-injury immune regulation; their metabolic programming is addressed later.

Resolution requires active clearance and reprogramming, not passive cytokine fading. Efferocytosis clears dying cells through recognition, engulfment and degradation, using find-me, eat-me and don't-eat-me signals to recruit and instruct phagocytes [45]. Effective efferocytosis suppresses pro-inflammatory cytokine release, increases anti-inflammatory signaling and supports tissue repair; defective clearance favors necrotic-core expansion, persistent inflammation, fibrosis or unstable remodeling [5, 45]. Failed efferocytosis therefore converts inflammation from a transient clearance program into a substrate- and phagocyte-dependent remodeling process.

## Metabolic reprogramming in cardiovascular inflammation: biochemical framework and interpretive principles

### Defining metabolic reprogramming in cardiovascular disease

In CVD, metabolic reprogramming refers to stress-responsive remodeling of substrate use, redox control, biosynthetic routing and metabolite signaling across cardiac and vascular cell types. Cells actively modify metabolic pathways and energy use in response to energy demand, proliferation pressure and hypoxia [46]; in the cardiovascular system, this response is interpreted through the demands of a specific compartment. A cardiomyocyte under ischemia or pressure overload faces ATP delivery, mitochondrial oxidation and ROS stress [1, 2]. Endothelial cells rely heavily on glycolysis in steady-state and injured myocardium; chronic injury may impair this glycolytic program and contribute to endothelial dysfunction [4]. Macrophages and T cells add another layer: glycolysis, tricarboxylic acid (TCA) cycle remodeling, lipid handling and amino-acid availability help determine cytokine production, efferocytosis and lineage behavior, not energy supply alone [4, 47]. Thus, a metabolic shift can protect one compartment acutely while amplifying inflammation in another. The following framework focuses on metabolic nodes that alter redox balance, inflammatory signaling or therapeutic interpretation.

### Core metabolic pathways relevant to cardiovascular inflammation

Glucose-centered metabolism illustrates this principle most clearly. Glycolysis converts glucose to pyruvate and yields rapid ATP; pyruvate then acts as a branch point, being reduced to lactate or entering mitochondria as acetyl-CoA for TCA/oxidative phosphorylation (OXPHOS) [48]. In immune and cardiovascular cells, however, routing matters more than pathway description. Glycolytic intermediates feed biosynthesis; glucose-6-phosphate can enter the pentose phosphate pathway (PPP), whose oxidative branch produces nicotinamide adenine dinucleotide phosphate (NADPH) and whose non-oxidative branch supports nucleotide precursor generation [49, 50]. In the cardiovascular setting, the glucose-6-phosphate dehydrogenase (G6PD)-dependent PPP is more relevant as a redox-buffering route because it helps maintain glutathione (GSH)-dependent control of ROS [51–53]. The tricarboxylic acid cycle (TCA cycle) is also more than an ATP pathway. Citrate and succinate can support inflammatory mediator production or hypoxia-inducible factor 1α (HIF-1α)-linked IL-1β programs [47, 54], and itaconate can restrain selected inflammatory outputs, at least in part through succinate dehydrogenase (SDH)-related control of succinate metabolism [4, 55]. Lactate belongs in the same framework because it can indicate glycolytic routing, signal through receptor-linked pathways such as G protein-coupled receptor 81 (GPR81), and, in selected cardiac contexts, enter chromatin-level regulation through histone lactylation [1, 11, 56].

Fatty-acid and ketone metabolism add an oxidation-capacity problem: benefit requires alignment among delivery, oxygen availability and mitochondrial capacity. The adult heart relies strongly on mitochondrial FAO, and fatty acids provide high ATP yield but at a higher oxygen cost and with greater dependence on intact oxidative capacity [1, 2]. FAO direction alone therefore provides an incomplete mechanistic readout. In heart failure with reduced ejection fraction (HFrEF), substrate preference varies with disease context and comorbidities; even nonischemic HFrEF can retain substrate flexibility when arterial substrate supply or workload changes [57]. Injury is more likely when lipid delivery and mitochondrial disposal become mismatched: excess lipid flux can be diverted toward acylcarnitines, ceramides, diacylglycerol (DAG) and other non-oxidative lipid products rather than efficient ATP production [1, 2, 57]. β-hydroxybutyrate (BHB) adds another layer because it can serve as an alternative mitochondrial fuel and, under selected conditions, as a signaling metabolite; its cardiovascular role remains promising but not settled [58, 59]. In humans, acute BHB infusion reduced myocardial glucose upta ke while increasing myocardial blood flow, confirming substrate-level effects without establishing anti-inflammatory mediation [60].

Amino-acid metabolism enters this review through inflammation, redox stress and mitochondrial dysfunction. Branched-chain amino acids/branched-chain α-keto acids (BCAA/BCKA) accumulation in HF is better read as impaired mitochondrial catabolic control, mechanistic target of rapamycin (mTOR)/ROS stress or defective branched-chain α-keto acids dehydrogenase (BCKDH) regulation than as simple amino-acid excess [2, 61]. In vascular disease, arginine metabolism links amino-acid flux to NO bioavailability, endothelial quiescence, leukocyte adhesion and platelet activity, whereas glycine, cysteine and glutamate support glutathione-dependent redox buffering [62]. In activated macrophages, argininosuccinate synthase 1-dependent citrulline depletion can additionally support pro-inflammatory polarization [63]. Glutamine and glutamate also contribute to anaplerosis and immune-cell metabolism [47]. Serine metabolism can also shape macrophage polarization through an insulin-like growth factor 1–p38 signaling route, indicating that amino-acid availability influences immune-cell state beyond anaplerosis [64]. These amino-acid nodes are included because they define inflammatory and redox mechanisms, not because they form a nutrition-style pathway survey.

Signaling metabolites provide the entry point for metabolic-inflammatory crosstalk. Succinate, itaconate and lactate connect glucose/TCA remodeling to inflammatory transcription, SDH-linked feedback control and receptor- or chromatin-associated signaling, respectively [4, 11, 47, 56]. BHB and ceramide/sphingosine-1-phosphate (S1P) extends this principle to ketone and lipid metabolism: BHB acts as a fuel-linked inflammatory modulator, whereas ceramide/S1P function mainly as lipid mediators, not simple pathway intermediates [1, 2, 59]. Basic metabolic pathways become inflammatory regulators when their intermediates are sensed by transcription factors, inflammasome machinery, lipid receptors or chromatin-regulatory systems.

### Interpreting metabolic evidence: flux, cell source and disease stage

Three methodological safeguards are central for CVD metabolism. First, metabolite abundance is not flux. Karlstaedt notes that a metabolite can accumulate because production rises, consumption falls or transporter activity changes; therefore, plasma concentration or tissue pool size does not resolve pathway activity [20]. Stable-isotope tracing and flux modeling are better suited to separate substrate availability from pathway use, although design and sampling remain critical [20]. Second, bulk tissue is not a cell source. The heart and vessel wall contain cardiomyocytes, endothelial cells, fibroblasts, resident macrophages, monocyte-derived macrophages, T cells and lymphatic cells; these compartments can move in opposite metabolic directions during the same disease stage [4, 54]. Single-cell maps of the adult human heart confirm this cellular diversity and reveal distinct transcriptional programs across cardiomyocytes, endothelial cells, fibroblasts and immune cells [65, 66]. Spatial transcriptomics, proteomics and metabolomics help localize molecular states in situ, but they still require functional validation [67, 68]. Other protocol-level variables, including anesthesia, may also affect hemodynamics and substrate use, although inconsistent reporting prevents their contribution from being assigned across the studies reviewed here. Third, injury timing rewrites pathway meaning. In early ischemic stress, glycolytic adaptation can support cardiomyocyte survival, as shown by PFK-related acute glycolytic responses and heat shock protein family A member 12 A (HSPA12A)-dependent glycolysis/lactylation signaling [11, 69, 70]. PPP activation belongs to the same acute-protection window mainly through redox control: L-2-hydroxyglutarate-driven PPP routing and G6PD-dependent glutathione buffering both points to NADPH/GSH-sensitive protection [51–53, 69]. Persistent glycolytic or lactate-linked signaling later shifts the question toward fibrosis, cytokine output or failed resolution [4, 47]. Similarly, FAO may support contractility when oxidation is matched to capacity, but lipid flux becomes injurious when it produces lipotoxic intermediates [2, 57]. Thus, the therapeutic question is not whether a pathway is activated, but whether the activated pathway belongs to the pathogenic compartment at the actionable disease stage. Without that assignment, metabolic activation remains a biomarker-level observation rather than a target.

## General molecular mechanisms of metabolic–inflammatory crosstalk

**Fig. 1:**
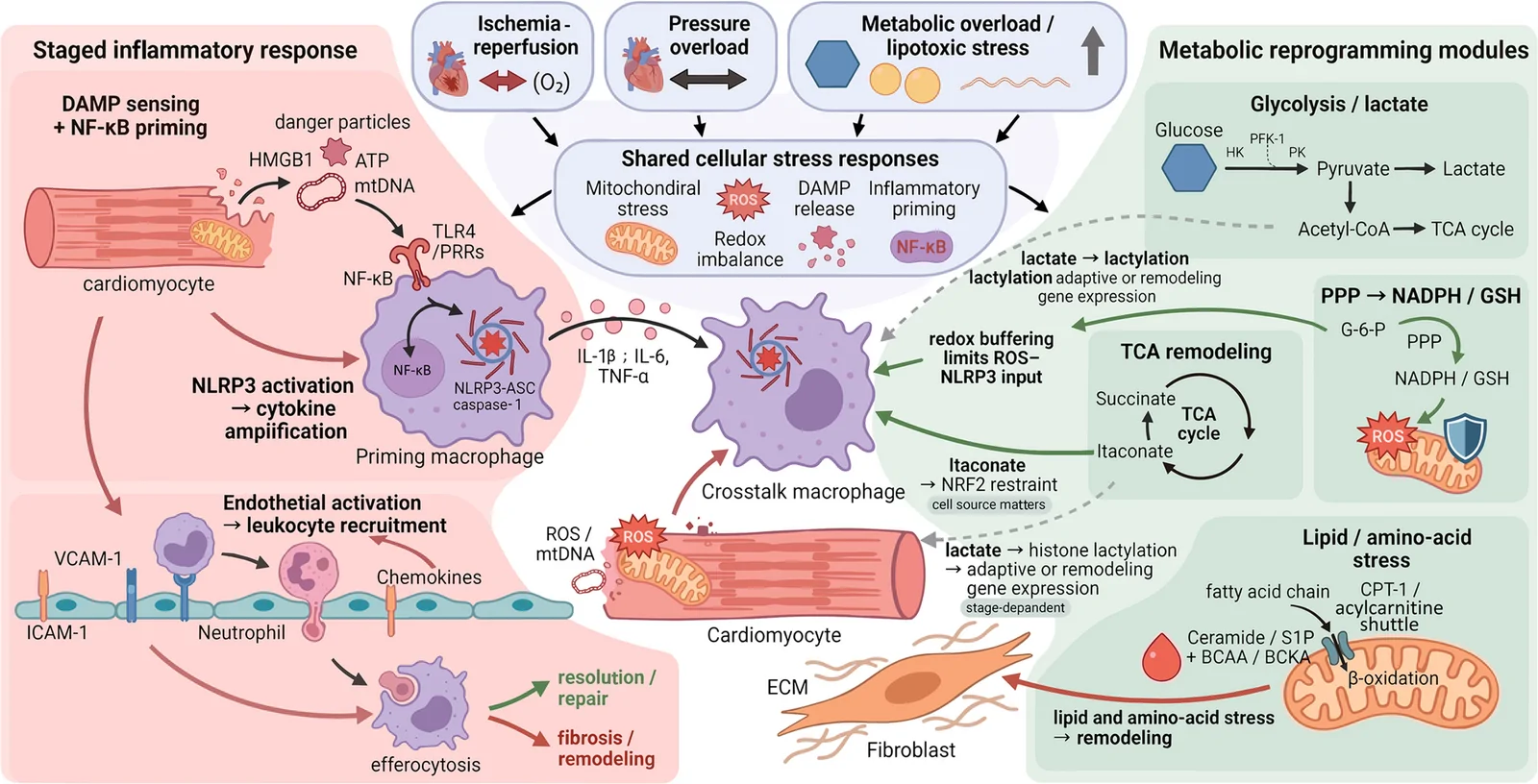
Overall interpretive framework of metabolism–inflammation crosstalk in cardiovascular disease. IRI, pressure overload and metabolic overload/lipotoxic stress converge on shared cellular stress responses, including mitochondrial stress, redox imbalance, DAMP release and inflammatory priming. The left panel outlines the staged inflammatory response described in Sect. "Core cardiovascular inflammatory pathways: from danger-signal sensing to chronic remodeling". DAMPs released from stressed cardiomyocytes activate PRRs/TLR4-dependent NF-κB priming in macrophages, thereby licensing NLRP3–ASC–caspase-1 inflammasome activation and downstream IL-1β, IL-6 and TNF-α amplification. Endothelial activation, VCAM-1/ICAM-1 expression and chemokine gradients organize leukocyte recruitment, after which efferocytosis and tissue repair compete with persistent inflammation and fibrotic remodeling. The right panel summarizes the metabolic routing required to interpret this inflammatory sequence. Glucose enters glycolysis through HK-, PFK-1- and PK-regulated steps to generate pyruvate, which can be reduced to lactate or converted to acetyl-CoA for TCA-cycle entry; G-6-P can also be diverted into the PPP to generate NADPH-dependent redox capacity. Long-chain fatty acids require CPT-1/acylcarnitine shuttle-dependent mitochondrial entry before β-oxidation, whereas BCAA catabolism generates BCKA intermediates that can enter mitochondrial metabolic networks [48–50, 61, 122, 187, 202, 215, 301–304]. The central crosstalk nodes highlight functional links rather than exhaustive pathways. PPP-derived redox buffering limits ROS-sensitive NLRP3 input; lactate links glycolytic flux to lactylation-associated adaptive or remodeling gene expression; TCA remodeling generates divergent signals, with succinate supporting HIF-1α–IL-1β inflammatory output and itaconate promoting NRF2-associated restraint; and lipid/amino-acid stress, including ceramide/S1P and BCAA/BCKA imbalance, connects mitochondrial injury to remodeling. These modules should be read in relation to the producing cell, disease stage, substrate routing and measurement layer, rather than as fixed protective or pathogenic signals. Abbreviations: ASC, apoptosis-associated speck-like protein containing a CARD; ATP, adenosine triphosphate; BCAA, branched-chain amino acid; BCKA, branched-chain α-keto acids; CoA, coenzyme A; CPT-1, carnitine palmitoyltransferase 1; DAMP, damage-associated molecular pattern; ECM, extracellular matrix; G-6-P, glucose-6-phosphate; GSH, glutathione; HIF-1α, hypoxia-inducible factor 1α; HK, hexokinase; HMGB1, high-mobility group box 1; ICAM-1, intercellular adhesion molecule 1; IL-1β, interleukin-1β; IL-6, interleukin-6; IRI, ischemia–reperfusion injury; mtDNA, mitochondrial DNA; NADPH, nicotinamide adenine dinucleotide phosphate; NF-κB, nuclear factor-κB; NLRP3, NOD-like receptor family pyrin domain-containing 3; NRF2, nuclear factor erythroid 2-related factor 2; PFK-1, phosphofructokinase-1; PK, pyruvate kinase; PPP, pentose phosphate pathway; PRRs, pattern-recognition receptors; ROS, reactive oxygen species; S1P, sphingosine-1-phosphate; TCA, tricarboxylic acid; TLR4, Toll-like receptor 4; TNF-α, tumor necrosis factor-α; VCAM-1, vascular cell adhesion molecule 1

Inflammatory responses in cardiovascular tissues are shaped by canonical immune signaling and by how cells sense, use and exchange metabolic substrates [47, 71]. The discussion follows three connected levels already used throughout the review: metabolic sensors, immune-cell metabolic reprogramming and signaling metabolites. Together, these levels explain how metabolic pressure is converted into inflammatory restraint, amplification or adaptation under specific cellular, stimulus and disease conditions. These relationships are integrated in Fig. 1, which links shared cellular stress responses to staged inflammatory activation and to the major metabolic reprogramming modules discussed below.

### Metabolic sensors regulating inflammation

Metabolism–inflammation crosstalk begins with cellular sensing before it becomes a visible inflammatory phenotype. AMP-activated protein kinase (AMPK), mechanistic target of rapamycin complex 1/2 (mTORC1/2), sirtuin 1/peroxisome proliferator-activated receptor γ coactivator 1α (SIRT1/PGC-1α), peroxisome proliferator-activated receptors (PPARs), nuclear factor erythroid 2-related factor 2 (NRF2), HIF-1α and NLRP3 respond to different metabolic pressures, including energy shortage, nutrient abundance, lipid overload, redox imbalance, hypoxia and mitochondrial danger signals [71–76]. These sensors function as interacting nodes that translate metabolic pressure into inflammatory restraint, amplification or adaptation, with outputs varying according to the responding cell, stimulus intensity and disease phase [77–79].

Energy stress provides a direct entry into this sensing network, and AMPK is one major node through which it is interpreted. Under energetic stress, AMPK limits anabolic energy consumption and supports mitochondrial and autophagic homeostasis in immune cells [71, 77]. In macrophages, this often aligns with weaker NF-κB-related inflammatory signaling and improved mitochondrial or autophagic control; AMPKα1 deficiency, by contrast, enhances lipopolysaccharide (LPS)-induced M1-like polarization and changes multiple metabolic enzymes after inflammatory stimulation [77, 80]. AMPK functions as a stimulus-sensitive brake on selected inflammatory metabolic programs, especially during acute pro-inflammatory stimulation, with its effect varying across immune and cardiovascular settings [77, 80]. The nicotinamide adenine dinucleotide (NAD⁺)–SIRT1–PGC-1α axis extends this adaptive response by linking energy stress to NAD⁺-dependent deacetylation, mitochondrial biogenesis and oxidative metabolic capacity [73]. Other sirtuin isoforms can act in a cell-specific manner; macrophage SIRT2 reduced atherosclerotic plaque formation in low-density lipoprotein receptor-deficient (*Ldlr −/−*) mice [81].

Nutrient sufficiency introduces a different problem: immune cells must decide whether to enter anabolic growth and effector programs. Nutrient and growth-factor sufficiency activate mTOR-dependent anabolic programs that support immune-cell activation, proliferation, cytokine production and effector function through glycolysis, protein translation, lipid synthesis and mitochondrial function [72, 82]. In macrophages, mTOR output follows the surrounding signal more than activation status alone. During interleukin-4/interleukin-13 (IL-4/IL-13)-induced alternative activation, the protein kinase B (Akt)–mTORC1–ATP citrate lyase (ACLY) axis helps convert glucose, fatty acid and glutamine inputs into acetyl-CoA for histone acetylation and selected M2-associated transcriptional programs [83]. ACLY is not restricted to alternative activation; in LPS-stimulated macrophages, it also supports selected pro-inflammatory outputs, including nitric oxide, ROS and prostaglandin E2 [84]. Under LPS/interferon-γ (IFN-γ) stimulation, amino-acid availability can instead support IL-1β-related inflammatory output through mTOR signaling [73]. mTOR operates as a nutrient-sensitive immunometabolic integrator, with output set by surrounding cytokines and substrates [72, 78].

Lipid and redox stress require a different set of sensors. PPARα, PPARβ/δ and PPARγ are lipid ligand-activated nuclear receptors that regulate FAO, lipid storage, glucose metabolism and immune-cell functional responses [74, 85]. In macrophages, PPAR signaling may support anti-inflammatory or repair-associated programs, but the effect varies with isoform, ligand, disease context and microenvironment; the lipid-sensing context sets the direction of the inflammatory effect [85, 86]. NRF2 provides the redox counterpart. In the canonical Kelch-like ECH-associated protein 1 (KEAP1)–NRF2 axis, oxidative stress releases NRF2 from KEAP1-mediated degradation, allowing NRF2 to induce antioxidant and detoxification-related genes [75, 87]. Itaconate creates a direct immunometabolic connection: by modifying KEAP1, itaconate activates NRF2 and converts a TCA-derived metabolite signal into an anti-inflammatory transcriptional program [88].

Once metabolic stress reaches hypoxia, mitochondrial injury or danger-signal release, HIF-1α and NLRP3 become more relevant. HIF-1α links hypoxia, glycolytic reprogramming and inflammatory transcription, especially interleukin 1 beta gene (*IL1B*)-related programs in activated immune cells [71, 79]. mTORC1 can support HIF-1α-dependent glycolysis and immune effector differentiation, while acute hypoxia in monocytes or macrophages integrates oxygen tension with ROS, Ca2⁺/protein kinase C signaling and NF-κB-related inflammatory cues [71, 79, 89]. HIF-1α therefore marks hypoxia-adapted glycolysis and *IL1B* transcription, whereas inflammasome activation requires separate evidence [76, 79, 90]. NLRP3 is placed further downstream: after priming, mitochondrial ROS (mtROS), mtDNA, ionic stress and organelle injury can promote inflammasome assembly, caspase-1 activation and IL-1β/IL-18 maturation [76]. Separating the two prevents conflating inflammatory gene transcription with mature cytokine release and pyroptotic output [79].

Metabolic sensors therefore form an interconnected regulatory network, not a checklist of markers. Stimulus intensity, exposure duration and responding cell type determine whether AMPK, mTOR, HIF-1α, NRF2 or NLRP3 restrain inflammation, amplify it or support adaptation. In the disease sections, sensor activation is therefore used as an entry point only when it can be linked to the source cell, injury phase and measured output.

### Immune-cell metabolic reprogramming

The upstream nodes discussed above can influence the metabolic programs of immune cells, enabling different cell types to develop distinct patterns of substrate utilization and functional phenotypes during activation, effector responses, resolution or repair [91]. Macrophages and the T helper 17/regulatory T cell (Th17/Treg) axis provide the main examples here, because they show how metabolic rewiring alters inflammatory output through defined immune-cell states and functions [47, 71, 92, 93].

In macrophages, classical pro-inflammatory stimulation by LPS/TLR4 and IFN-γ usually shifts cells toward higher glucose uptake and glycolysis, with parallel use of glycolytic branch pathways such as the PPP [47, 92, 94]. Faster ATP generation is only part of this shift. Glycolytic routing supplies biosynthetic intermediates and reducing power, while TCA-cycle remodeling gives several intermediates signaling functions [95]. The best-supported example remains the succinate–HIF-1α–IL-1β axis: in LPS-stimulated macrophages, succinate accumulation stabilizes HIF-1α and preferentially promotes IL-1β transcription, and pyruvate kinase M2 (PKM2) can further connect glycolytic state with HIF-1α-dependent inflammatory transcription [96–98]. The evidence therefore points to a selective IL-1β-linked program, in which glycolysis, PPP activity and TCA remodeling converge; TNF-α, IL-6 and tissue-adapted macrophage states do not map cleanly onto the same axis. Conversely, severe disruption of glycolytic flux can itself trigger inflammasome signaling and pyroptosis, underscoring that reduced glycolysis is not uniformly anti-inflammatory [99].

Mitochondria add another determinant to macrophage fate. In pro-inflammatory macrophages, mitochondria can remain active and be redirected toward SDH-dependent succinate oxidation, membrane-potential changes and mtROS generation, whereas itaconate can counter this route by inhibiting SDH and limiting succinate oxidation, mitochondrial respiration and selected inflammatory outputs [100, 101]. The same mitochondrial system can therefore support either inflammatory amplification or restraint, depending on how redox pressure and quality control are handled. When damaged mitochondria accumulate, mtROS and cytosolic or oxidized mtDNA can feed NLRP3 activation and IL-1β/IL-18 maturation; when mitophagy is effective, these danger signals are removed before the inflammasome loop becomes self-sustaining [102–105]. Thus, mitochondrial homeostasis is an active regulatory process that influences whether inflammation persists or resolves.

Macrophage lipid handling extends beyond FAO. Lipid load, cholesterol efflux, foam-cell formation and efferocytosis determine whether macrophages clear dying cells and support resolution or maintain tissue injury [94, 106, 107]. Efferocytosis can also depend on non-canonical glutamine transamination that sustains oxidative phosphorylation and redox buffering [108]. Apoptotic-cell-derived fatty acids can also support mitochondrial β-oxidation and electron-transport-chain activity, thereby promoting IL-10-associated reparative programming in efferocytic macrophages [109]. FAO/OXPHOS often accompanies repair-associated programs, but this association is conditional. In lipid-overloaded or inflammasome-primed settings, lipid metabolism may instead align with IL-1β-related inflammation. A functional reading is more useful here: macrophage lipid handling helps define whether the same cell contributes to debris clearance, inflammatory persistence or tissue repair.

**Fig. 2:**
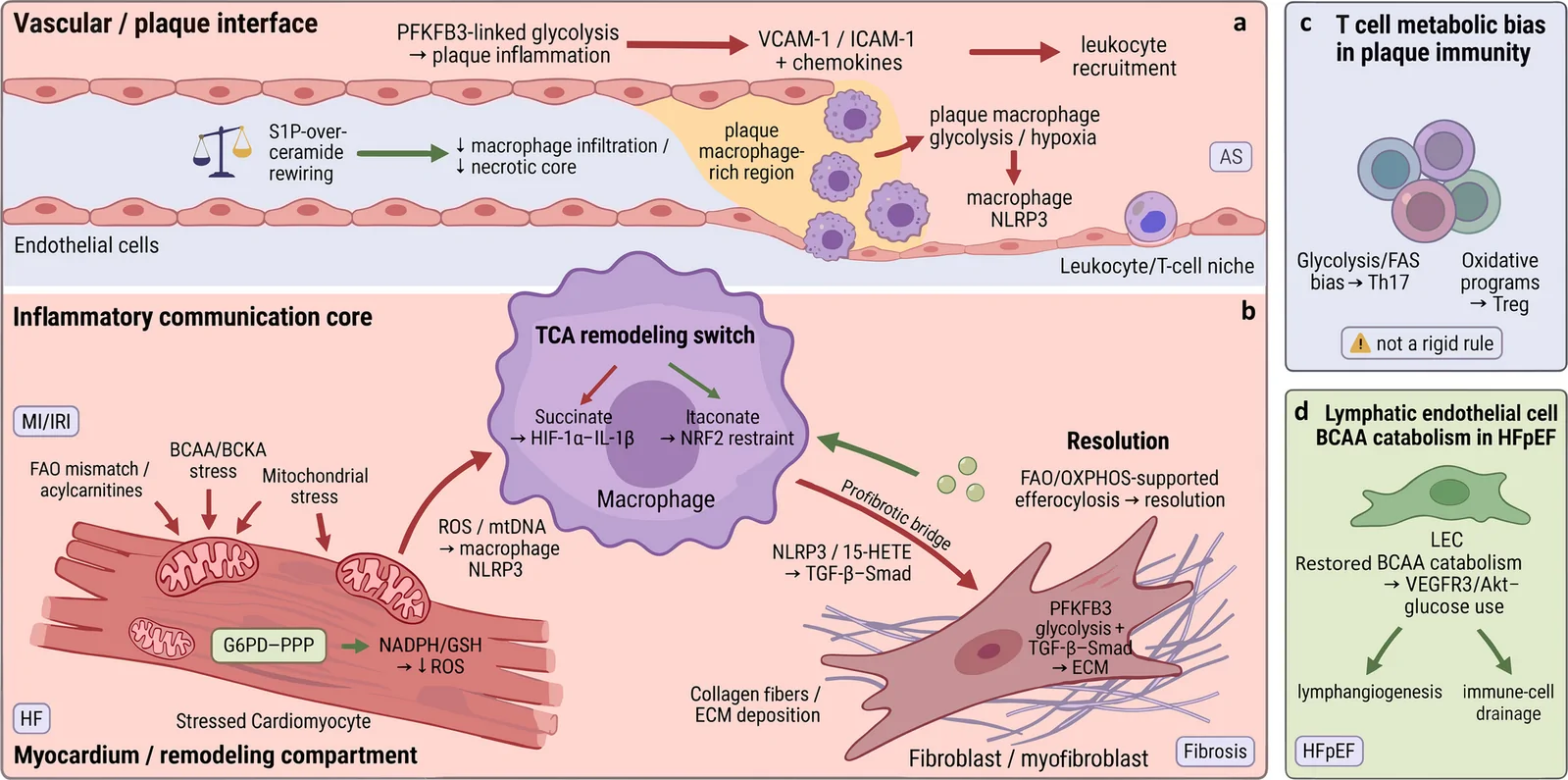
Cell–cell topology of metabolic-inflammatory crosstalk in cardiovascular disease. This figure maps spatial communication routes through which metabolic programs acquire inflammatory, reparative or remodeling-associated meaning in cardiovascular disease. **a** At the vascular/plaque interface, endothelial glycolytic activation, adhesion and chemokine cues, plaque macrophage hypoxia and S1P-favored ceramide/S1P rewiring shape leukocyte recruitment, macrophage-rich inflammation and plaque stability. **b** In the myocardium/remodeling compartment, stressed cardiomyocytes provide mitochondrial, redox and amino-acid stress cues that converge on macrophage NLRP3 activation, while macrophage TCA remodeling separates succinate–HIF-1α–IL-1β inflammatory output from itaconate–NRF2 restraint. This compartment also links macrophage-derived profibrotic signaling to fibroblast glycolysis, TGF-β–Smad activation, ECM deposition and FAO/OXPHOS-supported efferocytosis during resolution. **c** T-cell metabolic bias in plaque immunity is shown as a context-dependent balance between glycolysis/fatty acid synthesis-associated Th17 programs and oxidative metabolism-associated Treg programs. **d** In HFpEF, lymphatic endothelial BCAA catabolism supports VEGFR3/Akt-linked glucose use, lymphangiogenesis and immune-cell drainage. Abbreviations: 15-HETE, 15-hydroxyeicosatetraenoic acid; Akt, protein kinase B; AS, atherosclerosis; BCAA, branched-chain amino acid; BCKA, branched-chain α-keto acids; ECM, extracellular matrix; FAO, fatty acid oxidation; FAS, fatty acid synthesis; G6PD, glucose-6-phosphate dehydrogenase; GSH, glutathione; HF, heart failure; HFpEF, heart failure with preserved ejection fraction; HIF-1α, hypoxia-inducible factor 1α; ICAM-1, intercellular adhesion molecule-1; IL-1β, interleukin-1β; LEC, lymphatic endothelial cell; MI/IRI, myocardial infarction/ischemia–reperfusion injury; mtDNA, mitochondrial DNA; NADPH, nicotinamide adenine dinucleotide phosphate; NLRP3, NOD-like receptor family pyrin domain-containing 3; NRF2, nuclear factor erythroid 2-related factor 2; OXPHOS, oxidative phosphorylation; PFKFB3, 6-phosphofructo-2-kinase/fructose-2,6-bisphosphatase 3; PPP, pentose phosphate pathway; ROS, reactive oxygen species; S1P, sphingosine-1-phosphate; Smad, mothers against decapentaplegic homolog; TCA, tricarboxylic acid; TGF-β, transforming growth factor-β; Th17, T helper 17 cell; Treg, regulatory T cell; VCAM-1, vascular cell adhesion molecule-1; VEGFR3, vascular endothelial growth factor receptor 3

Macrophages illustrate how metabolic rewiring reshapes innate inflammatory output. In adaptive immunity, metabolism shapes lineage choice, especially within the Th17/Treg axis. After T cell receptor (TCR), costimulatory and cytokine signals, phosphoinositide 3-kinase (PI3K)–Akt, mTOR and liver kinase B1 (LKB1)–AMPK networks connect immune activation with substrate use and lineage fate [47, 71, 110]. Th17 differentiation is supported by glycolysis, HIF-1α and de novo fatty acid synthesis, whereas Treg programs are often associated with oxidative metabolism and lipid use [111–113]. That distinction is a useful starting point, but it becomes less clean in human or tissue-adapted T-cell states. Human Treg cells may also display glycolysis, mitochondrial respiration and FAO, although they appear less glycolysis-dependent than Th17 cells [114]. Th17/Treg metabolism therefore describes a bias in immune-cell programming, not a rigid substrate assignment [115]. This lineage tendency is represented in Fig. 2c as a metabolic bias rather than a rigid glycolysis-versus-oxidative-metabolism rule.

Immune-cell metabolic reprogramming becomes mechanistically informative only when linked to cell state and function. Glycolysis, TCA remodeling, mitochondrial quality control, lipid handling and T-cell substrate choice combine into adjustable programs [116]. Macrophage and T-cell metabolism should therefore be interpreted by function: the same glycolytic or oxidative program may support cytokine production, efferocytosis, antigen-experienced plaque inflammation or reparative remodeling depending on the disease compartment. Representative cell- and compartment-level relationships are mapped in Fig. 2, whereas Table 1 systematically compares their disease context, functional reading, remodeling output, and key interpretive cautions.

**Table 1:** Cell-type-specific interpretation of metabolic pathways in cardiovascular inflammation

Cell type/compartment	Metabolic node or program	Main disease context	Functional reading	Inflammatory or remodeling output	Key interpretive caution	Key refs
Cardiomyocyte	Acute glycolysis and HSPA12A-linked H3 lactylation	MI/IRI, early reperfusion	Adaptive within the acute injury window	Supports short-term energy adaptation, reduces ROS-associated injury, and promotes cardiomyocyte survival	Should not be extrapolated to persistent glycolysis, chronic inflammation, or late remodeling	[10, 11]
Cardiomyocyte/myocardium	PPP/G6PD/NADPH–GSH axis	MI/IRI and myocardial redox stress	Predominantly redox-buffering when linked to NADPH/GSH control	Limits ROS accumulation and supports redox-sensitive myocardial protection	More informative when tied to redox flux and GSH/GSSG control than to G-6-P abundance alone; cell source should be specified where possible	[51–53, 69]
Cardiomyocyte	FAO mismatch and acylcarnitine accumulation	MI/IRI, HF	Pathogenic when lipid delivery exceeds oxygen-dependent mitochondrial disposal	Impaired OXPHOS, mitochondrial hyperpolarization, ROS generation, and cell injury	FAO is not intrinsically harmful; injury reflects mismatch among lipid supply, oxygen availability, and mitochondrial oxidative capacity	[117–120]
Cardiomyocyte/myocardium	BCAA/BCKA catabolic imbalance	MI/IRI, HFrEF, stress-induced remodeling	Pathogenic or vulnerability-associated when catabolism is impaired	Mitochondrial dysfunction, ROS stress, lipid peroxidation, and increased ischemic susceptibility	BCAA/BCKA accumulation should not be interpreted as flux without compartment-specific catabolic evidence	[121–124]
Cardiomyocyte/hypertrophic myocardium	Pyruvate–lactate imbalance and lactate export	Stress-induced hypertrophic remodeling, HF models	Context-dependent; potentially pro-hypertrophic when persistent	Reduced mitochondrial pyruvate oxidation, lactate export, ROS stress, and hypertrophic growth	Clinical/genetic HCM should be separated from pressure-overload or metabolic-stress remodeling models	[17, 56, 125]
Macrophage	Glycolysis, PKM2, and succinate-linked TCA remodeling	Inflammatory macrophage activation; AS/hypoxic plaque as disease extension	Pro-inflammatory in activated macrophage states	HIF-1α-dependent IL-1β transcription and cytokine amplification	Evidence is strongest for IL-1β-linked transcription, not for all cytokines or all macrophage states	[13, 96, 97, 126]
Macrophage/innate immune cell	Mitochondrial damage, mtROS, and mtDNA	Sterile inflammatory activation, AS, post-MI inflammatory fibrosis	Pathogenic after priming when damaged mitochondria persist	NLRP3 activation, caspase-1 activity, and IL-1β/IL-18 maturation	HIF-1α-driven inflammatory transcription and mature inflammasome activation should remain mechanistically distinct	[103, 104, 127, 128]
Macrophage	Itaconate/ACOD1/NRF2 axis	AS, inflammatory restraint	Anti-inflammatory or resolution-supporting in selected contexts	NRF2-dependent restraint of inflammatory output and limitation of plaque inflammation	Itaconate is a regulatory macrophage signal, not a generic marker of plaque activation	[88, 101, 129]
Macrophage	Lipid handling, FAO/OXPHOS, and efferocytosis	AS, MI repair, chronic inflammation	Context-dependent; reparative during clearance but pathogenic in inflammasome-primed lipid overload	Efferocytosis, resolution, foam-cell persistence, or IL-1β-linked inflammation	FAO/OXPHOS should be interpreted by macrophage function rather than as a fixed M2-like signature	[130–135]
Plaque macrophage–endothelial compartment	PFKFB3-linked glycolysis	AS, plaque vulnerability, hypoxic plaque inflammation	Pathogenic when linked to glycolytic inflammatory activation	Leukocyte recruitment, intraplaque neovascularization, necrotic-core expansion, and plaque vulnerability	PFKFB3 signals in plaque macrophages and endothelial cells should not be assigned to one compartment without cellular evidence	[13, 126]
Endothelial cell	S1P-favored sphingolipid remodeling	Coronary AS, endothelial stress	Protective or adaptive in the endothelial context described	Reduced endothelial activation, macrophage infiltration, necrotic core, and plaque progression	Sphingolipid biology should be interpreted by lipid species and cell source, not as generic ceramide toxicity	[136]
Lymphatic endothelial cell	BCAA catabolism	HFpEF	Protective when intact	VEGFR3/Akt signaling, lymphangiogenesis, lymphatic integrity, and immune-cell drainage	HFpEF LEC BCAA biology should not be merged with cardiomyocyte BCAA stress	[137]
Fibroblast	PFKFB3-dependent glycolysis and TGF-β-linked fibrotic activation	Post-MI fibrosis, pressure-overload remodeling, myocardial fibrosis	Pro-fibrotic when persistent or coupled to TGF-β signaling	Myofibroblast activation, collagen deposition, and ECM expansion	Fibrosis should be assigned to fibroblast metabolic programs only when cellular evidence supports the source	[18, 19, 138, 139]
T cell compartment	Th17/Treg metabolic programming	AS, chronic plaque inflammation, immune regulation	Lineage- and state-dependent	Th17-skewed inflammatory programs or Treg-associated regulatory bias	Th17/Treg metabolism is a bias, not a rigid glycolysis-versus-FAO rule, especially in human tissue states	[111–115, 140, 141]

The functional reading refers to the cellular and disease context summarized in this review. The same pathway may shift from adaptive to pathogenic when flux direction, cell source, injury stage, or measurement layer changes

*Abbreviations:* *ACOD1* aconitate decarboxylase 1, *Akt* protein kinase B, *AS* atherosclerosis, *BCAA* branched-chain amino acids, *BCKA* branched-chain α-keto acids, *ECM* extracellular matrix, *FAO* fatty acid oxidation, *G-6-P* glucose-6-phosphate, *G6PD* glucose-6-phosphate dehydrogenase, *GSH* glutathione, *GSSG* oxidized glutathione, *HF* heart failure, *HFpEF* heart failure with preserved ejection fraction, *HFrEF* heart failure with reduced ejection fraction, *HCM* hypertrophic cardiomyopathy, *HIF-1α* hypoxia-inducible factor 1α, *HSPA12A* heat shock protein family A member 12 A, *IL-1β* interleukin-1β, *IL-18* interleukin-18, *IRI* ischemia–reperfusion injury, *LEC* lymphatic endothelial cell, *MI* myocardial infarction, *mtDNA* mitochondrial DNA, *mtROS* mitochondrial reactive oxygen species, *NADPH* nicotinamide adenine dinucleotide phosphate, *NLRP3* NOD-like receptor family pyrin domain-containing 3, *NRF2* nuclear factor erythroid 2-related factor 2, *OXPHOS* oxidative phosphorylation, *PFKFB3* 6-phosphofructo-2-kinase/fructose-2,6-bisphosphatase 3, *PKM2* pyruvate kinase M2, *PPP* pentose phosphate pathway, *ROS* reactive oxygen species, *S1P* sphingosine-1-phosphate, *TCA* tricarboxylic acid, *TGF-β* transforming growth factor-β, *Th17* T helper 17 cell, *Treg* regulatory T cell, *VEGFR3* vascular endothelial growth factor receptor 3

### Signaling metabolites and lipid mediators

The metabolic programs described above also generate metabolites that carry information beyond pathway activity. The section focuses on metabolites that function beyond pathway-intermediate status: succinate and itaconate from TCA remodeling, lactate from glycolytic flux, BHB from ketone metabolism, and ceramide/S1P from sphingolipid remodeling. The key variable is which sensor, enzyme, receptor or chromatin process is engaged in that cellular condition.

Among TCA-derived metabolites, succinate and itaconate illustrate two opposing outputs of TCA remodeling. In LPS-activated macrophages, succinate accumulation can stabilize HIF-1α and support IL-1β transcription, making succinate a link between TCA remodeling and inflammatory gene expression [96]. The claim should remain narrow: the evidence is strongest for IL-1β-related transcription, not for all cytokines. Itaconate shifts the same discussion in the opposite direction. By inhibiting SDH, itaconate limits succinate oxidation, mitochondrial respiration and selected inflammatory outputs; through KEAP1 modification, itaconate or its derivatives can also activate NRF2-dependent antioxidant and anti-inflammatory transcription [88, 101, 142]. Endogenous ACOD1-derived itaconate and membrane-permeable itaconate derivatives should be interpreted separately, because genetic loss-of-function and derivative-based experiments interrogate distinct exposure and electrophilic mechanisms [88, 101, 143]. Beyond itaconate signaling, fumarate and dimethyl fumarate can modify GSDMD through succination and thereby limit pyroptotic pore formation [144]. The same TCA node can therefore point toward inflammatory transcription or inflammatory restraint, depending on whether succinate accumulation, SDH activity or itaconate–NRF2 signaling dominates.

Lactate converts glycolytic flux into extracellular, enzymatic and chromatin-level signals. It can act outside the producing cell, interact with enzyme- or receptor-linked pathways, and enter chromatin regulation through histone lysine lactylation. In obese adipose tissue, adipocyte-derived lactate can reinforce macrophage IL-1β through a prolyl hydroxylase domain protein 2–HIF-1α route [145]. Lactate–GPR81 signaling points in the opposite direction in other inflammatory models, where it has been linked to reduced NF-κB/NLRP3-related output [146–148]. Histone lactylation adds a third route: lactate-derived lysine lactylation (Kla) can mark late inflammatory macrophages and induce homeostatic or repair-associated genes such as arginase 1 (Arg1) and vascular endothelial growth factor A (Vegfa) [149]. Route and setting determine lactate biology. Its relevance is that altered glycolysis can become extracellular signaling, enzyme sensing or chromatin-level transcriptional regulation.

BHB supports a more circumscribed claim. As the major circulating ketone body, BHB may restrain NLRP3 activation in selected experimental systems; Youm et al. linked this effect to reduced K⁺ efflux, ASC oligomerization and IL-1β/IL-18 release [150]. Fasting, ketogenic diets and exogenous ketones also change insulin, lipolysis, fatty acid supply and energy balance, so their inflammatory effects cannot be attributed to BHB alone [151]. Human acute ketone supplementation has not consistently reproduced NLRP3 suppression in LPS-stimulated monocytes [152]. BHB should be interpreted as one ketone-body signal capable of modulating NLRP3 under selected conditions; ketosis itself remains a broader metabolic state.

Sphingolipids extend this discussion into lipid signaling. Ceramide, sphingosine and S1P are membrane-associated lipids, but they also influence cell death, inflammation, immune responses, vascular function and cardiac remodeling [153]. Ceramide is most relevant here as a lipotoxic inflammatory signal: in obesity-related models, it can promote NLRP3-dependent caspase-1 activation and IL-1β/IL-18 release [154]. S1P biology is more receptor- and stimulus-dependent. Sphingosine kinase 1/2 (SPHK1/2)-derived S1P can contribute to NLRP3 and *IL1B* priming, while sphingosine-1-phosphate receptor (S1PR)-dependent effects vary with receptor subtype and stimulus context [155, 156]. Lipid species, enzymes and receptor subtypes provide safer analytical units than a single inflammatory lipid class.

These metabolites are most informative when the producing cell and sensing mechanism are identifiable. Succinate and itaconate capture opposing inflammatory consequences of macrophage TCA remodeling, lactate links glycolytic flux to receptor- and chromatin-level signaling, BHB identifies a ketone-sensitive but context-dependent NLRP3 regulatory point, and ceramide/S1P distinguishes lipotoxic inflammasome activation from enzyme- and receptor-dependent sphingolipid signaling.

**Fig. 3:**
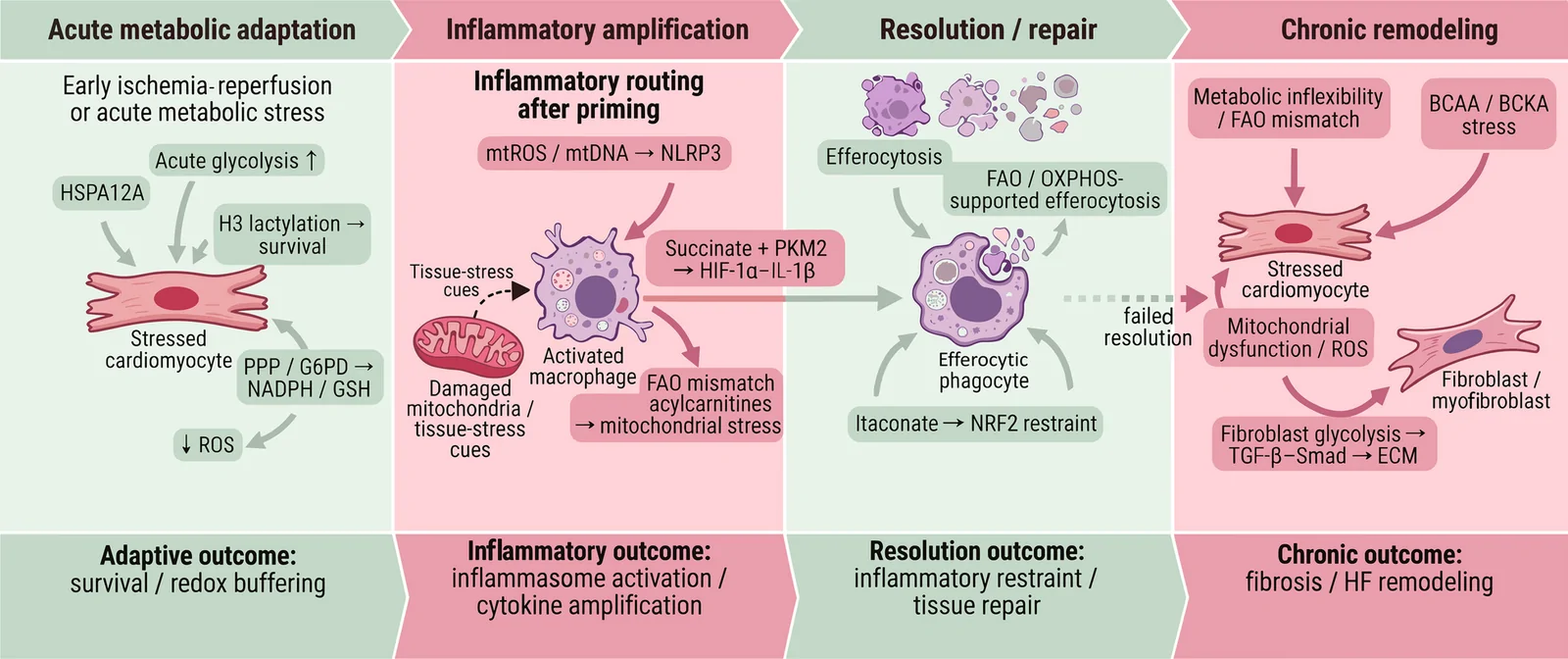
Stage-dependent interpretation of metabolic-inflammatory programs across cardiovascular injury and remodeling. This figure illustrates how timing and cellular source determine the interpretation of metabolic-inflammatory programs during cardiovascular injury and remodeling. During acute metabolic adaptation, early ischemia–reperfusion or acute metabolic stress can engage cardiomyocyte-protective glucose programs, including HSPA12A-associated glycolysis and histone H3 lactylation, acute glycolytic support, and PPP/G6PD-dependent NADPH–GSH redox buffering. These programs are linked to adaptive outcomes such as survival and ROS limitation. During inflammatory amplification, damaged mitochondria, mtROS, mtDNA and tissue-stress cues promote macrophage activation and NLRP3-related signaling, while succinate- and PKM2-associated HIF-1α–IL-1β signaling and FAO mismatch/acylcarnitine accumulation may facilitate inflammasome activation under primed conditions, cytokine amplification and mitochondrial stress. During resolution/repair, efferocytosis, FAO/OXPHOS-supported phagocyte function and itaconate–NRF2 restraint shift the response toward inflammatory resolution and tissue repair. Failed resolution provides a bridge to chronic remodeling, where metabolic inflexibility, FAO mismatch, BCAA/BCKA stress, mitochondrial dysfunction and fibroblast glycolysis cooperate with TGF-β–Smad signaling to promote ECM deposition, fibrosis and HF remodeling. Abbreviations: BCAA, branched-chain amino acid; BCKA, branched-chain α-keto acids; ECM, extracellular matrix; FAO, fatty acid oxidation; G6PD, glucose-6-phosphate dehydrogenase; GSH, glutathione; H3, histone H3; HF, heart failure; HIF-1α, hypoxia-inducible factor-1α; HSPA12A, heat shock protein family A member 12 A; IL-1β, interleukin-1β; mtDNA, mitochondrial DNA; mtROS, mitochondrial reactive oxygen species; NADPH, nicotinamide adenine dinucleotide phosphate; NLRP3, NOD-like receptor family pyrin domain containing 3; NRF2, nuclear factor erythroid 2-related factor 2; OXPHOS, oxidative phosphorylation; PKM2, pyruvate kinase M2; PPP, pentose phosphate pathway; ROS, reactive oxygen species; TGF-β, transforming growth factor-β

Viewed across disease progression rather than as isolated pathways, these mechanisms form a stage-dependent framework: acute metabolic adaptation may support survival and redox buffering, mitochondrial and TCA stress can amplify inflammation, successful efferocytosis supports resolution, and failed resolution favors chronic fibrotic remodeling (Fig. 3). The sequence is interpretive rather than obligatory because the same pathway can change meaning with cell type and disease context.

## Metabolic–inflammatory crosstalk in specific cardiovascular diseases

**Fig. 4:**
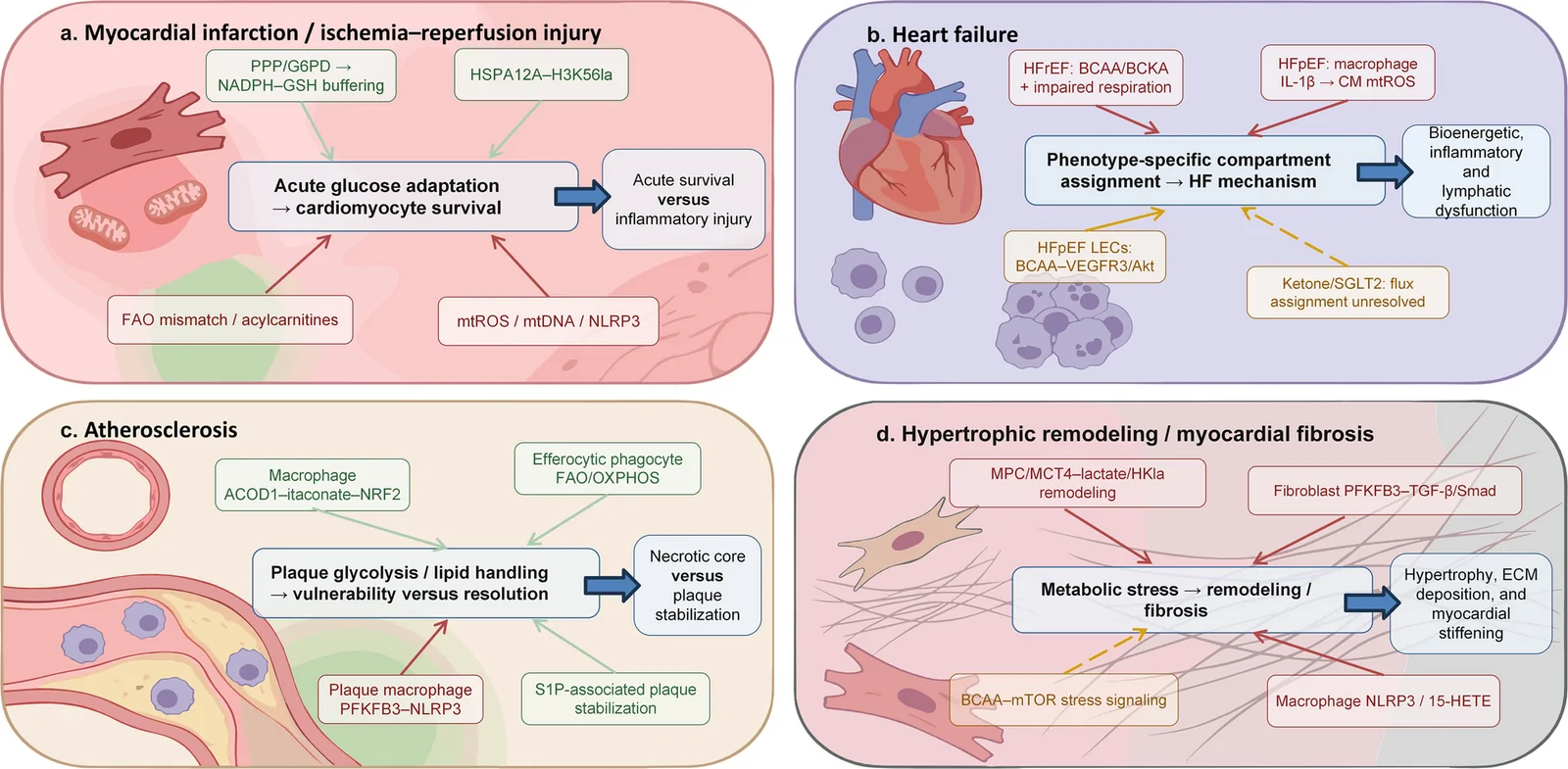
Disease-prioritized integrative model of metabolic-inflammatory crosstalk in cardiovascular disease. The figure organizes metabolic-inflammatory mechanisms according to disease phenotype, dominant cellular compartment, priority metabolic module and disease output. Green modules denote adaptive, reparative or stabilizing programs; red modules denote inflammatory, injury-promoting or profibrotic programs; and yellow modules denote phenotype-dependent or incompletely resolved mechanisms whose interpretation requires additional information on cell compartment, flux direction or disease setting. Solid arrows indicate assigned mechanistic links within the depicted disease context, dashed arrows indicate conditional or unresolved links that should not yet be interpreted as fixed causal routes. **a** In myocardial infarction/ischemia–reperfusion injury, acute glucose adaptation in cardiomyocytes is positioned as a survival module shaped by PPP/G6PD-dependent NADPH–GSH buffering and HSPA12A–H3K56la signaling, whereas FAO mismatch, acylcarnitine accumulation and mtROS/mtDNA/NLRP3 activation shift the same injury context toward inflammatory damage. Timing determines whether this biology is therapeutically actionable. **b** In heart failure, HFrEF is linked to BCAA/BCKA stress and impaired respiration, whereas HFpEF includes macrophage IL-1β–CM mtROS signaling, LEC BCAA–VEGFR3/Akt metabolism and unresolved ketone/SGLT2 flux assignment; the two phenotypes should not be merged as a single metabolic-inflammatory state. **c** In atherosclerosis, plaque outcome is framed as a balance between glycolysis/lipid-handling programs that promote vulnerability and resolution programs that support stabilization. Macrophage PFKFB3–NLRP3 favors inflammatory plaque vulnerability, whereas ACOD1–itaconate–NRF2 signaling, efferocytic phagocyte FAO/OXPHOS and S1P-associated signaling support resolution or stabilization. Plaque stability is therefore more informative than pathway direction alone. **d** In hypertrophic remodeling/myocardial fibrosis, metabolic stress is linked to remodeling and fibrosis through MPC/MCT4–lactate/histone lactylation signaling, fibroblast PFKFB3–TGF-β/Smad activation, BCAA–mTOR stress signaling and macrophage NLRP3/15-HETE input. The output is hypertrophy, ECM deposition and myocardial stiffening, while stress-induced remodeling remains distinct from clinical or genetic HCM. *Abbreviations:* 15-HETE, 15-hydroxyeicosatetraenoic acid; ACOD1, aconitate decarboxylase 1; Akt, protein kinase B; BCAA, branched-chain amino acids; BCKA, branched-chain α-keto acids; CM, cardiomyocyte; ECM, extracellular matrix; FAO, fatty acid oxidation; G6PD, glucose-6-phosphate dehydrogenase; GSH, glutathione; H3K56la, histone H3 lysine 56 lactylation; HCM, hypertrophic cardiomyopathy; HF, heart failure; HFpEF, heart failure with preserved ejection fraction; HFrEF, heart failure with reduced ejection fraction; HKla, histone lysine lactylation; HSPA12A, heat shock protein family A member 12 A; IL-1β, interleukin-1β; LECs, lymphatic endothelial cells; MCT4, monocarboxylate transporter 4; MPC, mitochondrial pyruvate carrier; mtDNA, mitochondrial DNA; mtROS, mitochondrial reactive oxygen species; mTOR, mechanistic target of rapamycin; NADPH, nicotinamide adenine dinucleotide phosphate; NLRP3, NOD-like receptor family pyrin domain-containing 3; NRF2, nuclear factor erythroid 2-related factor 2; OXPHOS, oxidative phosphorylation; PFKFB3, 6-phosphofructo-2-kinase/fructose-2,6-bisphosphatase 3; PPP, pentose phosphate pathway; S1P, sphingosine-1-phosphate; SGLT2, sodium–glucose cotransporter 2; Smad, mothers against decapentaplegic homolog; TGF-β, transforming growth factor-β; VEGFR3, vascular endothelial growth factor receptor 3

To preserve disease and compartment specificity, the evidence is organized into four disease-prioritized modules—MI/IRI, heart failure, atherosclerosis, and hypertrophic/fibrotic remodeling—using a common framework of dominant cell compartment, priority metabolic module, disease output, and interpretation boundary (Fig. 4).

### Myocardial infarction and ischemia–reperfusion injury: acute metabolic stress and sterile inflammation

MI and IRI comprise temporally and spatially distinct metabolic states. Ischemia, early reperfusion, inflammatory amplification and repair impose different demands on cardiomyocytes, endothelial cells, infiltrating leukocytes and fibroblasts. Spatial multi-omic analysis of human MI and single-cell profiling of experimental post-MI hearts resolve distinct injury, inflammatory, stromal and reparative compartments [157, 158]. Consistent with this heterogeneity, CD45^+^ immune-cell populations change dynamically after MI, while macrophage metabolic and phenotypic states vary with cellular origin and time after infarction [159–162]. Macrophages occupy a central position in this transition by contributing to inflammation, debris clearance, angiogenesis and scar maturation [132, 163]. Later-stage studies identify triggering receptor expressed on myeloid cells 2-high (TREM2-high) macrophages in reparative regions, and a TREM2–SLC25A53 metabolic program has been linked to their reparative function [164, 165]. Together, these findings frame the post-MI response as a transition from acute metabolic adaptation and inflammatory injury toward either reparative reprogramming or maladaptive remodeling.

#### Acute glycolytic adaptation and PPP-dependent redox buffering

The functional consequences of glucose routing differ between ischemia, early reperfusion and later remodeling. Increasing phosphofructokinase (PFK) expression during the acute phase can mitigate hypoxia-induced myocardial damage, and L-2-hydroxyglutarate can shift glucose metabolism toward the PPP, lowering ROS and reducing MI extent [69, 70]. Beltran et al. further separated glycolysis-dominant from OXPHOS-dominant states in a glucose/galactose H9C2 model; glucose-supported glycolysis lowered ROS and reduced simulated IRI cell death, although this model did not test immune inflammation or in vivo remodeling [10]. Yu et al. provided a more reperfusion-specific example: HSPA12A maintained HIF-1α-dependent glycolytic gene expression and histone H3 lysine 56 (H3K56) lactylation, and its protection was lost when glycolysis or H3 lactylation was inhibited [11]. These findings place early glycolysis within a cardiomyocyte survival program during acute injury; later MI progression requires a different interpretation.

Accumulation of individual glycolytic intermediates does not necessarily indicate protective glycolytic flux. During IRI, glucose-6-phosphate (G-6-P) can rise markedly [70], and high G-6-P has been linked to hexokinase 2 (HK2) detachment from the voltage-dependent anion channel, a mitochondrial event associated with cardiomyocyte death in ischemic hearts [117, 166, 167]. PPP activity is more directly tied to redox buffering. G6PD-dependent glutathione control limits ROS-sensitive cardiac dysfunction during IRI, whereas G6PD deficiency or cardiomyocyte G6PD suppression lowers the reduced/oxidized glutathione (GSH/GSSG) ratio, increases ROS and weakens contractility [51–53, 69]. Thus, PPP/G6PD defines early cardiomyocyte redox protection and should not be generalized to all inflammatory cells.

#### Mitochondrial stress and inflammatory cell death

Mitochondrial stress is the point where metabolic imbalance most clearly turns inflammatory. During ischemia, fatty acid (FA) metabolism becomes uncoupled from mitochondrial oxidation, long-chain FAs accumulate, and carnitine palmitoyltransferase 1 (CPT-1) generates long-chain acylcarnitines that hypoxic mitochondria cannot fully process [117–119]. These intermediates can inhibit OXPHOS, promote mitochondrial hyperpolarization and increase ROS production [117, 118]. Bugger and Pfeil place this ROS burst within both acute IRI injury and later post-MI remodeling, making mitochondrial ROS a recurring mediator of acute injury and later remodeling [120].

NLRP3 gains explanatory value in MI/IRI through specific metabolic-stress routes. One route is phosphoglycerate mutase family member 5 (PGAM5)-mitochondrial antiviral signaling protein (MAVS)-NLRP3 signaling: membrane-associated ring-CH-type finger 2 (MARCH2) promotes K48-linked PGAM5 degradation, reducing PGAM5-MAVS co-condensation, NLRP3 activation and cardiomyocyte pyroptosis in IRI models [168]. Hyperuricemia provides another context, where uric acid (UA) increases ROS, NLRP3-related pyroptotic proteins, lactate dehydrogenase (LDH) release and cardiomyocyte death; ROS scavenging or NLRP3 inhibition only partly reverses these effects [169]. Thus, NLRP3 amplifies selected ROS- or mitochondrial danger-linked states without serving as the final common pathway of all MI/IRI metabolism.

Lipid-derived inflammatory injury also extends beyond acylcarnitines. After MI, ceramide synthesis genes and several ceramide species increase; acid ceramidase–modified mRNA lowers ceramide accumulation and is associated with less cardiomyocyte death, fewer pro-inflammatory neutrophils, improved function and smaller scars [12]. This supports a sphingolipid-inflammatory route in post-MI injury, with interpretation still requiring attention to ceramide species and disease stage across ischemia, reperfusion and repair.

#### Amino acid–lipid crosstalk in ischemic injury

The FAO literature in IRI is mixed because benefit requires substrate oxidation to remain matched to respiratory capacity. Peroxisome proliferator-activated receptor β/δ (PPARβ/δ) activation can preserve mitochondrial respiratory function, lower ROS and reduce infarct size during myocardial IRI [170]. By contrast, chronic BCAA/BCKA accumulation in adult ventricular cardiomyocytes increases PPARα-dependent FAO, lipid peroxidation and IRI susceptibility [121]. Mitochondrial protein phosphatase 2 C (PP2Cm) lowers BCAA accumulation and protects against ischemic injury [122]. The decisive variable is whether oxygen-limited mitochondria can use the incoming lipid flux efficiently: coordinated oxidation can support recovery, whereas BCAA/BCKA-driven lipid flux can feed oxidative injury. In a recent MI study, glutamine supplementation improved LV function, whereas glutaminase inhibition worsened it; neither intervention measurably altered infarct-macrophage gene expression or bioenergetics [171]. MI/IRI therefore fits a staged model of metabolic injury. Early glycolysis supports acute energy adaptation [69, 70], while PPP/G6PD activity protects mainly through NADPH/GSH-dependent redox buffering [51–53]. The injurious phase is more heterogeneous: glycolytic overload and acylcarnitine-linked mitochondrial stress connect substrate imbalance to ROS production [117–119, 166, 167], ceramide accumulation adds a sphingolipid-inflammatory route after MI [12], and BCAA/BCKA-driven FAO can increase lipid peroxidation and IRI susceptibility [121, 122]. Accordingly, MI/IRI spans the acute-adaptation and inflammatory-amplification portions of the stage-dependent framework in Fig. 3, with later outcome determined by the balance between resolution and maladaptive remodeling. The disease-specific integration of these adaptive and injurious MI/IRI modules is summarized in Fig. 4a.

### Heart failure: metabolic inflexibility, mitochondrial dysfunction and chronic inflammation

HF comprises distinct metabolic–inflammatory phenotypes rather than a single pattern of substrate failure. HFrEF has been associated with altered FA availability and FAO-related remodeling, whereas heart failure with preserved ejection fraction (HFpEF) metabolomic studies indicate a different myocardial lipid profile, including lower myocardial FA levels relative to HFrEF [172, 173]. Human myocardial studies further associate inflammation with extracellular-matrix accumulation and diastolic dysfunction in HF with normal or preserved ejection fraction, while circulating IL-6 is associated with HFpEF severity and prognosis [174, 175]. These observations define phenotype-level inflammatory associations but do not by themselves identify the responsible cellular mechanism.

Cell-resolved studies identify several non-equivalent mechanisms. In high-fat diet (HFD)/diabetes-associated diastolic dysfunction, macrophage-derived IL-1β increases cardiomyocyte mtROS, and IL-1 receptor blockade, macrophage depletion or mtROS scavenging improves diastolic function [176]. Single-cell profiling further identifies metabolically stressed cardiac macrophage states in dyslipidemia-associated diastolic dysfunction [177]. In a separate HFpEF compartment, impaired BCAA catabolism in lymphatic endothelial cells disrupts VEGFR3/Akt signaling, lymphangiogenesis and immune-cell drainage [137]. Peripheral blood mononuclear cells (PBMCs) bioenergetic dysfunction in advanced HFrEF extends the metabolic–inflammatory phenotype beyond the myocardium to the systemic immune compartment [178]. These compartment-specific findings motivate separate consideration of myocardial substrate inflexibility, BCAA handling and ketone/sphingolipid signaling in the following subsections.

#### Substrate inflexibility and mitochondrial redox stress

Metabolic inflexibility provides a more precise starting point than pathway up- or down-regulation alone. Pyruvate dehydrogenase kinase 4 (PDK4)-mediated pyruvate dehydrogenase (PDH) inhibition limits TCA-cycle entry, while electron-transport-chain impairment and reduced TCA cycle activity can increase reliance on glycolysis when oxidative metabolism fails [179]. This may be compensatory, but it also signals reduced flexibility: the failing myocardium becomes less able to shift among glucose oxidation, FAO, ketone oxidation and amino-acid catabolism according to oxygen supply and substrate availability. Redox stress enters as soon as impaired fuel use damages mitochondria. Mitochondrial dysfunction, lipid peroxidation, mtDNA damage and lower ATP production mark the point at which impaired fuel use begins to reshape inflammatory and remodeling signals [2, 172, 180].

The FAO literature is stage-dependent. Advanced HF is often associated with reduced FAO and weaker oxidative metabolic programs [2, 172, 180, 181]. Changes in PPARα/PGC-1α signaling and CPT-1-linked FA transport provide part of this transcriptional and transport-level explanation [173, 182, 183]. However, PPARα activation improves remodeling only when restored oxidative metabolism matches mitochondrial capacity [184]. Oxidative programs are helpful only when mitochondria can use the incoming substrate efficiently; otherwise, FA supply can become lipotoxic. NAD^+^ extends this discussion beyond cardiomyocytes. Lower NAD^+^ or NAD^+^/NADH ratios have been reported in HF, and impaired NAD-biosynthesis gene expression has been observed in HFpEF cardiac tissue [82, 185, 186]. Zhou et al. moved this argument into immune cells: PBMCss from stage D HFrEF patients showed reduced maximal respiration and higher inflammatory gene expression, while mitochondrial damage-associated molecular pattern (MitoDAMP)-induced IL-6 impaired complex I activity. Nicotinamide riboside (NR) improved PBMCs respiration and reduced cytokine gene expression in a very small HF cohort, pointing to NAD^+^ availability as a possible connection between immune-cell bioenergetics and systemic inflammation [178].

#### BCAA catabolism and phenotype-specific HF remodeling

BCAA metabolism carries mechanistic information in HF after compartment assignment. In HFrEF, BCAA and BCKA accumulation has been linked to impaired mitochondrial function and ROS-related injury [61, 123, 187–189]. Li et al. added a mitochondrial regulatory axis: myocardial 3-mercaptopyruvate sulfurtransferase (3-MST) was reduced in human HFrEF, and 3-MST deficiency in transverse aortic constriction (TAC) mice increased myocardial BCAA accumulation, impaired mitochondrial respiration and ATP synthesis, and worsened cardiac and vascular dysfunction. BT2 or the hydrogen sulfide (H_2_S) donor JK-1 rescued part of this phenotype, placing 3-MST-derived mitochondrial H_2_S upstream of BCAA catabolic control and making BCAA accumulation more than a passive metabolomic marker [124].

In HFpEF, the stronger cell-type-specific evidence comes from lymphatic endothelial cells. Guo et al. identified impaired BCAA catabolism as a prominent metabolic signature of HFpEF cardiac lymphatic endothelial cells (LECs) [137]. BCAA catabolic defects reduced vascular endothelial growth factor receptor 3 (VEGFR3) membrane availability, weakened Akt signaling, impaired glucose use and suppressed lymphangiogenesis; LEC-specific branched-chain α-keto acids dehydrogenase kinase (Bckdk) deletion preserved lymphatic integrity and protected against obesity/hypertension-associated HFpEF [137]. The relevance of this finding is strongest in HFpEF, where it explains why cardiomyocyte energetics alone cannot account for the phenotype. The macrophage data from Liu et al. align with this compartment-specific pattern from another cellular interaction: metabolic stress in HFD/diabetic HFpEF can become a macrophage–cardiomyocyte inflammatory circuit through IL-1β and mtROS, without proving that BCAA metabolism directly drives macrophage IL-1β [176]. This compartment boundary is illustrated in Fig. 2d, where lymphatic endothelial BCAA catabolism is kept distinct from myocardial BCAA/BCKA stress.

#### Ketone and sphingolipid signaling in HF remodeling

Ketone bodies and sphingolipids converge on inflammatory remodeling through distinct fuel and lipid-mediatory routes. Ketone use may be adaptive when FAO and glucose oxidation are inefficient. In a TAC model, chronic BHB elevation was associated with less cardiac dysfunction, remodeling and inflammatory-marker expression, and BHB reduced NLRP3 activation in isolated hearts [190]. The evidence supports a pressure-overload anti-inflammatory signal but does not generalize all ketone elevation as therapeutic. Goedeke et al. add a metabolic boundary: dapagliflozin and acute BHB infusion produced different myocardial substrate patterns, and in failing rat hearts dapagliflozin increased ketone and FA oxidation, reduced pyruvate oxidation, improved mitochondrial redox and lowered oxidative stress [191].

Lipid signaling contributes most clearly through ceramide/S1P-related lipotoxic inflammation. Ji et al. found increased total and very-long-chain ceramides in advanced human HF myocardium and serum, partial reversibility after unloading, and reduced ventricular remodeling, fibrosis and macrophage content after serine palmitoyltransferase (SPT) inhibition [192]. In diabetic mice, AdipoRon lowered cardiac free fatty acid (FFA), triglycerides and TLR4-related ceramide accumulation, increased acid ceramidase activity, shifted the ceramide/S1P balance, and was associated with less M1-dominant inflammation, apoptosis, oxidative stress and fibrosis [193]. This evidence places ceramide/S1P between saturated lipid overload and inflammatory remodeling. HF therefore enters the therapeutic section as a phenotype-specific targeting problem: the relevant target may be cardiomyocyte substrate use, immune-cell respiration, lymphatic endothelial BCAA handling or lipid-derived inflammatory remodeling [137, 176, 178, 190, 192, 193]. This phenotype-specific assignment of myocardial, immune, lymphatic, and lipid-remodeling mechanisms is summarized in Fig. 4b.

### Atherosclerosis: vascular immunometabolism and plaque inflammation

Atherosclerosis operates as a vascular immunometabolic disease in which endothelial cells react to disturbed flow and lipid stress, macrophages link glucose and lipid handling with inflammatory activation and efferocytosis, vascular smooth muscle cells (VSMCs) shape cap stability, and T cells add adaptive immune pressure [13, 136, 140, 194]. Systemic metabolic stress can also sustain vascular inflammation through hyperglycemia-induced trained immunity and metabolic-syndrome-associated bone-marrow activation [195, 196]. Single-cell studies of human plaques resolve distinct macrophage, lymphocyte, endothelial and VSMC states within the same lesion [197, 198]. Single-cell analyses further identify VSMC state transitions that can influence fibrous-cap composition and plaque stability [199, 200]. The central question is which cell type relies on a specific pathway to drive endothelial activation, necrotic-core formation, defective resolution, plaque instability or thrombosis [13, 126, 136, 201].

#### Endothelial and macrophage glycolysis in plaque vulnerability

In AS, inducible glycolysis becomes mechanistically relevant when plaque macrophage or endothelial programs track with vulnerability. A more pronounced glycolytic phenotype has been reported in AS lesions, and partial inhibition of glycolysis can reduce plaque development and intraplaque neovascularization, although plaque composition is not always substantially altered [202, 203]. PFKFB3 offers a more disease-specific signal. In human plaques, high PFKFB3 expression was enriched mainly in macrophages and endothelial cells from vulnerable carotid plaques and correlated with necrotic-core area in advanced coronary plaques. In *Ldlr −/− *mice, PFK158 reduced PBMCs glycolysis, necrotic-core size and intraplaque apoptosis, while increasing fibrous-cap thickness [13, 204]. Because PFK158 is a pharmacological intervention, the macrophage-only mechanism remains unresolved. Even so, these data place inducible glycolysis closer to plaque vulnerability than to plaque size alone. The consequence of PFKFB3 suppression can also extend beyond reduced glycolysis: in a separate atherosclerosis model, it increased macrophage-derived VEGF-C and lymphangiogenesis [205].

Hypoxic plaque macrophages provide one possible mechanism linking glycolysis to inflammasome activation. Independent genetic evidence also indicates that macrophage HIF-1α promotes atherosclerotic lesion development [206]. Publicly available data from Wang et al. show that PFKFB3 accumulates in human plaques and colocalizes with macrophage/NLRP3 signals. In apolipoprotein E-deficient (*ApoE −/−*) mice and hypoxic bone marrow-derived macrophages (BMDMs), PFK158 or HIF-1α/PFKFB3 blockade reduced glucose uptake, glycolytic flux, NLRP3, caspase-1 and IL-1β [126]. Because the available evidence is strongest in hypoxic BMDMs and PFK158-treated mouse plaques, these data support a hypoxia–PFKFB3–macrophage inflammasome route within plaques. Plaque metabolism also involves non-macrophage immune compartments. IFN-γ-producing CD8⁺ and CD4⁺ T cells are major immune populations in human carotid plaques, and plaque single-cell T cell receptor sequencing (scTCR-seq) identified clonally expanded effector CD4^+^ T cells consistent with recent antigen engagement [140, 207]. These observations support discussing plaque metabolism alongside antigen-experienced T-cell inflammation, while avoiding a purely autoimmune interpretation of AS. The plaque-specific endothelial-macrophage and T-cell relationships underlying these observations are summarized in Fig. 2a.

#### Lipid handling, inflammatory resolution and plaque stability

Macrophage lipid handling can contribute either to resolution or to inflammatory amplification, depending on context. During efferocytosis, macrophage FAO and OXPHOS help sustain necrotic-cell clearance and inflammatory resolution [130, 208]. In an inflammasome-primed setting, however, lipid oxidation may align more closely with IL-1β production, and FAO inhibition has been reported to reduce macrophage NLRP3 activation and IL-1β release while slowing plaque formation [131]. FAO here functions as a marker of macrophage state, with its interpretation determined by efferocytosis, inflammasome priming and plaque stage.

Different lipid species also generate distinct inflammatory phenotypes. Within the plaque-stability discussion, palmitic acid is most relevant in type 2 diabetes mellitus (T2DM). In diabetic plaques, palmitic acid (PA)-induced macrophage delta-like ligand 4 (Dll4) promoted VSMC senescence, reduced collagen synthesis and increased plaque vulnerability. Human cohort and mouse data further supported this PA–macrophage Dll4–VSMC senescence axis [194]. Sphingolipid findings point in another direction. In coronary AS, endothelial neurite outgrowth inhibitor-B (NOGO-B) deletion maintained sphingolipid remodeling toward S1P rather than ceramide, producing an atheroprotective endothelial transcriptional signature with thicker caps, smaller necrotic cores and reduced macrophage infiltration [136]. Plaque lipid metabolism therefore separates by both cell type and lipid species: saturated FA stress may destabilize plaques through macrophage–VSMC crosstalk, whereas endothelial S1P-favored sphingolipid rewiring may reflect a protective adaptation.

#### Metabolite signals linking plaque inflammation to atherothrombotic risk

Metabolite signaling influences atherosclerotic disease through both local plaque mechanisms and systemic regulation of thrombotic responsiveness. Itaconate is the clearest macrophage-related example. Aconitate decarboxylase 1 (ACOD1) and itaconate increased during atherogenesis; myeloid *Acod1* deletion aggravated inflammation, expanded a specific M1-like macrophage subset and enlarged lesions, whereas 4-octyl itaconate reduced high-cholesterol-induced inflammation and AS through an NRF2-dependent anti-inflammatory response [129]. In this setting, itaconate functions as a macrophage inflammatory brake, not a generic marker of plaque activation. Together with the glycolytic, lipid-handling, and efferocytic mechanisms described above, these plaque-stabilizing and plaque-destabilizing routes are integrated in Fig. 4c.

At a different biological scale, circulating metabolites can modify thrombotic risk without directly reflecting plaque-cell metabolism. Phenylacetylglutamine (PAGln) shifts the focus from local plaque metabolism to gut microbiota-dependent thrombo-inflammatory signaling. PAGln has been associated with CVD and incident major adverse cardiovascular events (MACE), enhanced platelet responsiveness and thrombosis potential, and signaling through adrenergic receptors, including α2A, α2B and β2 receptors [201]. PAGln increases platelet reactivity and thrombotic susceptibility but does not directly reflect metabolic activity within the plaque. It therefore extends the discussion from local plaque inflammation to the systemic regulation of thrombosis.

### Cardiac hypertrophic remodeling and myocardial fibrosis: metabolic stress, inflammatory remodeling and ECM deposition

This section treats three related but non-equivalent forms of cardiac growth: clinical/genetic HCM, stress-induced pathological hypertrophic remodeling and adaptive/physiological hypertrophy. The term HCM is used only for patient tissue, HCM cohorts or clearly defined genetic HCM evidence [14–17]. Single-nucleus profiling of human DCM and HCM further shows that disease subtype and pathogenic variants reshape both cell composition and transcriptional state [209, 210]. Transverse aortic constriction/abdominal aortic constriction (TAC/AAC), chronic hypoxia, trimethyl-5-aminovaleric acid (TMAVA)/FAO suppression, BCAA-mTOR activation and mitochondrial pyruvate carrier/monocarboxylate transporter 4 (MPC/MCT4)-related pyruvate-lactate imbalance are described as stress-induced pathological hypertrophic remodeling [70, 123, 125, 211–216]. Exercise-related growth and short-term compensatory growth are treated as adaptive or physiological hypertrophy [211, 212, 217]. Fibrosis is included because pressure overload, MI and metabolic stress can shift the discussion from cardiomyocyte growth toward interstitial ECM remodeling. During this transition, cardiomyocytes may provide early metabolic signals, fibroblasts translate injury into collagen deposition, and macrophage inflammasome activity can strengthen the fibrotic component of remodeling [18, 19, 128, 218, 219].

#### Cardiomyocyte metabolic rewiring in hypertrophic remodeling

A common cardiomyocyte response in hypertrophic remodeling is a shift away from FA oxidation and toward glycolysis [211], but this shift splits into adaptive and pathological programs. In pressure overload, fructose-2,6-bisphosphate/phosphofructokinase-1 (F-2,6-BP/PFK-1)-driven glycolysis can support an adaptive response [212], and Yes-associated protein (YAP)-related Warburg metabolism has been linked to compensatory cardiomyocyte growth instead of pathological remodeling [217]. Chronic hypoxia or sustained metabolic stress gives the same glycolytic direction a different meaning, placing it closer to pathological hypertrophic remodeling [70, 211, 212]. Clinical HCM is a separate disease category: HCM tissues show higher free FAs, lower acylcarnitines, reduced β-oxidation enzyme expression and greater glucose/ketone utilization compared with donor tissue [14–16]. The separation also matters for FAO-related transcriptional evidence. PPARs regulate fatty acid uptake and oxidation [220], but the reported reduction of PPARα and downstream FAO genes comes from TAC/AAC-associated remodeling not direct HCM patient evidence.

Lactate-related remodeling gives this substrate shift a more persistent output. In hypertrophy and HF, reduced mitochondrial pyruvate import through MPC and increased MCT4-dependent lactate export define an early pyruvate-lactate imbalance [125]. MPC loss was sufficient to induce hypertrophy and HF in adult mouse hearts, whereas MCT4 inhibition reduced hypertrophic phenotypes in both cells and mice [125]. Histone lysine lactylation provides an additional chromatin-level route: glucose metabolism and lactate synthesis influence histone lysine lactylation (HKla), exogenous lactate increases HKla and promotes myocardial hypertrophy, while 2-deoxy-D-glucose (2-DG) lowers lactate production, reduces HKla and limits hypertrophy [56]. In this context, lactate marks the point at which altered glycolytic flux can be redirected into gene-regulatory growth programs.

#### Fibroblast glycolysis, transforming growth factor-β (TGF-β) signaling and ECM deposition

Fibrosis follows a cellular logic that differs from cardiomyocyte growth. PKM2 can worsen cardiac fibrosis through TGF-β/Smad2/3 and Janus kinase 2/signal transducer and activator of transcription 3 (Jak2/Stat3) signaling, and PKM2 inhibition reduces myofibroblast proliferation in vitro [70, 221, 222]. The stronger in vivo anchor is fibroblast-specific TGF-β signaling. In pressure-overload fibrosis, deletion of transforming growth factor-β receptor 1/2 (Tgfbr1/2) or Smad3 in fibroblast lineages markedly reduced fibrosis, whereas Smad2 deletion had little effect; receptor-level deletion also changed the hypertrophic response, suggesting fibroblast-cardiomyocyte communication beyond canonical Smad3 signaling [18]. This fits the broader fibrosis model in which acute myofibroblast formation supports repair, while persistent activation stiffens the ventricle and maintains ECM deposition [218].

Fibroblast metabolism gives this pathway a more direct metabolic foundation. In post-MI cardiac fibrosis, TGF-β1-stimulated cardiac fibroblasts showed higher glycolytic activity, lactate accumulation and a shift in the nicotinamide adenine dinucleotide (NADH/NAD^+^) redox ratio. PFKFB3 was upregulated and stabilized through OTU deubiquitinase 4 (OTUD4)-mediated deubiquitylation, while 3-(3-pyridinyl)−1-(4-pyridinyl)−2-propen-1-one (3PO) or *Pfkfb3* knockdown reduced α-smooth muscle actin (α-SMA), collagen expression, fibrosis and functional deterioration [19]. A related post-MI study highlights why timing is important: delayed, lower-dose 2-DG reduced collagen deposition and fibroblast activation, whereas immediate high-dose 2-DG increased rupture-related mortality [138]. The timing contrast suggests that early repair may require glycolysis, while later excessive fibroblast glycolysis helps sustain a profibrotic state. This timing dependence corresponds to the transition from repair-supporting metabolism to the chronic-remodeling end of the framework in Fig. 3.

#### Inflammatory input into fibrotic remodeling

Macrophage-derived exosomes provide an additional route of intercellular communication after MI. Exosomes from inflammatory macrophages can impair angiogenesis, whereas miR-155-containing exosomes suppress fibroblast proliferation and enhance fibroblast inflammatory signaling [223, 224]. Macrophages provide the inflammatory arm of fibrotic remodeling. In MI, NLRP3 activation peaked early and occurred mainly in macrophages at day 3. Macrophage-conditioned signaling promoted fibroblast-to-myofibroblast transition through a 15-hydroxyeicosatetraenoic acid (15-HETE)-mediated Smad pathway, and 15-HETE was elevated in MI mouse hearts and human MI/acute coronary syndrome (ACS) samples [128]. This evidence is most directly relevant to post-MI inflammatory fibrosis; its value here is showing how inflammasome activity can be translated into fibroblast activation and ECM deposition. Clinical/genetic HCM remains a separate category: patient-based single-nucleus and spatial transcriptomic data indicate multilineage remodeling involving cardiomyocytes, fibroblasts, immune cells and vascular cells, with TGF-β-related communication in fibrotic regions [17]. Asymmetric dimethylarginine (ADMA) may also contribute to impaired NO production and diastolic dysfunction in clinical HCM, whereas BCAA-mTOR signaling fits better as a metabolic stress-related hypertrophic remodeling, with direct HCM evidence still lacking [123, 187, 216, 225]. The cardiomyocyte-macrophage-fibroblast communication routes that connect metabolic stress to inflammatory and fibrotic remodeling are integrated in Fig. 2b. Figure 4d preserves the corresponding interpretation boundary by separating stress-induced hypertrophic/fibrotic remodeling from clinical or genetic HCM.

### Cross-disease integration: shared nodes, context dependency and unresolved questions

Across the cardiovascular conditions discussed above, the effects of glycolysis, fatty-acid oxidation, redox metabolism and amino-acid metabolism vary with cell type and disease stage. Atrial fibrillation (AF) adds a setting in which metabolic changes intersect with electrical, inflammatory and fibrotic remodeling.

Circulating TCA-cycle intermediates have been associated with incident AF. Elevated citrate, aconitate, succinate and malate were associated with AF risk, while aconitate, isocitrate and malate were also correlated with incident HF [226]. These circulating associations do not establish altered TCA-cycle flux in atrial cardiomyocytes. More direct evidence comes from experimental manipulation of citrate synthase. In angiotensin II (Ang II)-infused atria, citrate synthase expression was reduced, whereas cardiomyocyte-specific citrate synthase overexpression increased OXPHOS complex I–V expression and ATP production, reduced oxidative stress and attenuated atrial remodeling and AF vulnerability [227].

Inflammatory remodeling in AF also differs by cellular source. Cardiomyocyte NLRP3 activation altered Ca2⁺ handling, atrial effective refractory period, ion-channel expression and AF inducibility, linking inflammasome activity directly to the electrical substrate [228]. A separate inflammatory–fibrotic circuit involves recruited macrophages. In human AF atria and HOMER mice, expansion of secreted phosphoprotein 1-positive (SPP1⁺) macrophages was associated with fibroblast activation, collagen deposition and AF burden [229]. These findings distinguish cardiomyocyte-intrinsic inflammasome signaling from macrophage–fibroblast crosstalk in atrial remodeling.

Glycolysis has different consequences across these disease settings. During early MI/IRI, cardiomyocyte glycolysis and PPP/G6PD activity can support ATP production and NADPH/GSH-dependent redox control. By contrast, persistent PFKFB3-dependent glycolysis in plaque cells or cardiac fibroblasts is associated with inflammatory activation, plaque vulnerability or extracellular-matrix deposition [10, 11, 13, 19]. BCAA, GSH and TCA-cycle intermediates become mechanistic only after cell source and measurement layer are defined: BCAA/BCKA-driven FAO in IRI, lymphatic endothelial BCAA defects in HFpEF and platelet BCAA catabolism in thrombosis are shared substrate classes, but mechanistically separate processes [137, 188, 230, 231]. Similarly, glutathione metabolism has a more direct role in cellular redox buffering, while circulating glutamate and glutamine-to-glutamate measures are better interpreted as risk-associated metabolic markers unless their relationship to tissue flux is established [232, 233].

These comparisons also show why measurement methods cannot be treated as interchangeable. Plasma metabolomics, bulk-tissue assays, single-cell or spatial profiling and tracer-based flux studies address different questions [20, 234]. Taken together, Fig. 4A–D integrates the disease-specific evidence according to dominant cell compartment, priority metabolic module, disease output, and interpretation boundary. Table 2 extends this cross-disease comparison by summarizing how variation in cell source, disease stage, flux direction, and measurement layer can produce apparently contradictory metabolic findings.

**Table 2:** Context-dependent and apparently contradictory metabolic findings in metabolism–inflammation crosstalk

Metabolic node/pathway	Apparent contradiction	Adaptive/protective setting	Pathogenic/maladaptive setting	Key explaining variables	Interpretive conclusion	Key refs
Glycolysis in cardiomyocytes, macrophages and fibroblasts	Glycolysis can support acute cardiomyocyte survival yet accompany vascular inflammation or fibrotic remodeling	During acute MI/IRI, cardiomyocyte glycolytic routing supports survival, including HSPA12A-linked H3K56 lactylation	Plaque macrophage–endothelial PFKFB3 glycolysis associates with vulnerability; fibroblast PFKFB3 glycolysis sustains profibrotic activation	Cell identity; injury stage; glycolytic flux; ATP/redox demand versus cytokine or ECM output	Assign glycolysis to the relevant cell compartment and stage rather than treating it as uniformly protective or harmful	[10, 11, 13, 19, 138, 161]
PPP/G6PD/NADPH–GSH axis	PPP/G6PD redox protection may be confused with G-6-P-linked ischemic injury	In acute cardiomyocyte IRI, G6PD-dependent PPP supports NADPH/GSH buffering and limits ROS-sensitive dysfunction	G-6-P accumulation and HK2 detachment represent a distinct mitochondrial injury mechanism; no generic pathogenic PPP/G6PD state is asserted	PPP flux versus G-6-P abundance; G6PD activity versus broader redox biology; timing and cellular compartment	Interpret PPP/G6PD primarily as an early cardiomyocyte redox-protective route; do not infer PPP pathogenicity from G-6-P accumulation	[51–53, 69, 70, 117, 166, 167]
FAO/OXPHOS and lipid handling	Oxidative lipid metabolism may support resolution or generate lipotoxic injury	Macrophage FAO/OXPHOS can support efferocytosis, dead-cell clearance and inflammatory resolution	In oxygen-limited cardiomyocytes, long-chain acylcarnitines accumulate, impair OXPHOS and increase ROS; in inflammasome-primed macrophages, lipid oxidation may align with IL-1β-linked inflammation	Substrate load; oxygen supply; mitochondrial capacity; efferocytosis status; inflammasome priming	Distinguish matched oxidative use from lipid-overload mismatch and resolution metabolism	[117–120, 130, 131, 208, 235, 236]
Succinate/PKM2/HIF-1α–IL-1β versus mtROS/mtDNA–NLRP3	Glycolytic–TCA remodeling is often conflated with inflammasome activation	Hypoxia-adapted glycolytic routing may occur without inflammasome assembly; *IL1B* transcription alone does not establish NLRP3 activation	In activated macrophages, succinate/PKM2 promote HIF-1α-dependent *IL1B* transcription; following priming, mtROS/mtDNA facilitate NLRP3 assembly and cytokine maturation	Transcription versus cytokine maturation; priming versus activation; macrophage state; mitochondrial injury	Separate *IL1B* transcription from inflammasome assembly, caspase-1 activation and mature IL-1β release	[76, 79, 96, 97, 102–104]
Itaconate/ACOD1/NRF2 axis	Itaconate is often presented as uniformly anti-inflammatory despite distinct endogenous and derivative-based evidence	In macrophage-centered AS, ACOD1–itaconate signaling and 4-OI treatment reduce selected inflammatory outputs through SDH/NRF2-linked mechanisms	No generic pathogenic counterpart is defined in this review	Endogenous ACOD1–itaconate versus derivative treatment; macrophage source; exposure conditions; SDH/NRF2 engagement	Interpret ACOD1–itaconate signaling in its experimental context rather than as a uniform anti-inflammatory program	[88, 100, 101, 129, 237]
Lactate/lactylation	Lactate-linked signaling can accompany acute survival or chronic hypertrophic remodeling	During acute cardiomyocyte IRI, HSPA12A-linked H3K56 lactylation is part of a survival-associated glycolytic program	In hypertrophic remodeling, lactate/histone lactylation supports growth programs; fibroblast lactate accumulation accompanies profibrotic glycolysis without proving a lactylation-mediated fibrotic mechanism	Injury stage; cell source; lactate accumulation versus HKla; receptor-, enzyme- or chromatin-mediated route	Interpret lactate/lactylation by route and setting; do not equate lactate accumulation with lactylation-mediated fibrosis	[11, 19, 56, 125, 238]
BCAA/BCKA metabolism	BCAA-catabolic impairment is linked to distinct HF phenotypes and cell compartments	In HFpEF LECs, intact BCAA catabolism supports VEGFR3/Akt signaling, glucose utilization and lymphangiogenesis	In HFrEF myocardium, BCAA/BCKA accumulation associates with mitochondrial dysfunction and ROS injury; in HFpEF, LEC defects impair lymphatic drainage	HFrEF versus HFpEF; myocardium versus LECs; abundance versus catabolic flux; mitochondrial versus lymphatic endpoint	Assign BCAA/BCKA findings to the responsible compartment and HF phenotype before inferring mechanism	[61, 123, 124, 137, 187–189]
Stress-induced hypertrophic remodeling versus clinical/genetic HCM	HCM terminology may merge genetic disease with pressure-overload or metabolic-stress models	In early pressure-overload remodeling, glycolytic or Warburg-like programs may support compensatory growth	Chronic hypoxia, BCAA–mTOR activation and MPC/MCT4-related pyruvate–lactate imbalance represent stress-induced remodeling rather than clinical/genetic HCM	Etiology; genetic status; model type; disease stage; hypertrophy versus fibrosis endpoint	Reserve clinical/genetic HCM for defined HCM evidence; classify TAC/AAC and metabolic-stress models separately	[14–17, 70, 123, 125, 212, 217]
Measurement layer and evidence assignment	Different evidence layers can make the same metabolic node appear contradictory	Higher-confidence interpretation: tracer flux, cell-specific perturbation, spatial/single-cell localization, or phenotype-linked evidence	Overinterpretation risk: plasma or bulk abundance is treated as cell source, flux direction or causal inflammatory function	Bulk versus single-cell; plasma versus tissue; abundance versus flux; sampling time; association versus perturbation	Use measurement data to delimit mechanism; assign flux, source and pathogenicity only when evidence supports them	[20, 21, 32–34, 67, 162, 234, 239–241]

For the PPP/G6PD, succinate/PKM2/HIF-1α–IL-1β, itaconate/ACOD1/NRF2 and measurement-layer entries, the listed studies illustrate why pathway direction, sampling layer and experimental model can produce apparently divergent conclusions

*Abbreviations:* *4-OI* 4-octyl itaconate, *AAC* abdominal aortic constriction, *ACOD1* aconitate decarboxylase 1, *Akt* protein kinase B, *AS* atherosclerosis, *BCAA* branched-chain amino acids, *BCKA* branched-chain α-keto acids, *ECM* extracellular matrix, *FAO* fatty acid oxidation, *G-6-P* glucose-6-phosphate, *G6PD* glucose-6-phosphate dehydrogenase, *GSH* glutathione, *HCM* hypertrophic cardiomyopathy, *HF* heart failure, *HFpEF* heart failure with preserved ejection fraction, *HFrEF* heart failure with reduced ejection fraction, *HIF-1α* hypoxia-inducible factor 1α, *HK2* hexokinase 2, *HKla* histone lysine lactylation, *HSPA12A* heat shock protein family A member 12 A, *IL1B* interleukin 1 beta gene, *IL-1β* interleukin-1β, *IRI* ischemia–reperfusion injury, *LEC* lymphatic endothelial cell, *MCT4* monocarboxylate transporter 4, *MI* myocardial infarction, *MPC* mitochondrial pyruvate carrier, *mtDNA* mitochondrial DNA, *mtROS* mitochondrial reactive oxygen species, *mTOR* mechanistic target of rapamycin, *NADPH* nicotinamide adenine dinucleotide phosphate, *NLRP3* NOD-like receptor family pyrin domain-containing 3, *NRF2* nuclear factor erythroid 2-related factor 2, *OXPHOS* oxidative phosphorylation, *PFKFB3* 6-phosphofructo-2-kinase/fructose-2,6-bisphosphatase 3, *PKM2* pyruvate kinase M2, *PPP* pentose phosphate pathway, *ROS* reactive oxygen species, *SDH* succinate dehydrogenase, *TAC* transverse aortic constriction, *TCA* tricarboxylic acid, *VEGFR3* vascular endothelial growth factor receptor 3

## Therapeutic targets

### Therapeutic rationale and evidence boundaries

Therapeutic translation should start from the disease-driving cell population and the patients in whom the pathway is active. A candidate target needs evidence that the pathway is active, responds to intervention and can be modulated with a plausible safety margin; otherwise, it may remain useful for phenotyping or risk stratification but not yet function as a therapeutic mechanism. Galectin-3 illustrates this distinction: experimental data support a causal contribution to inflammatory-fibrotic remodeling, whereas HFpEF patient data primarily support biomarker and prognostic associations rather than therapeutic targetability [240, 241].

Clinical anti-inflammatory trials illustrate why patient selection and pathway engagement matter. In the Canakinumab Anti-inflammatory Thrombosis Outcomes Study (CANTOS), IL-1β blockade reduced recurrent cardiovascular events in stable post-MI patients selected for elevated high-sensitivity CRP (hsCRP), without lowering low-density lipoprotein cholesterol (LDL-C) [242]. In the Cardiovascular Inflammation Reduction Trial (CIRT), low-dose methotrexate in stable AS with diabetes or metabolic syndrome did not reduce IL-1β, IL-6, CRP, or cardiovascular events [243]. This contrast supports a targeted inflammatory-risk model rather than nonspecific anti-inflammatory suppression. The Colchicine Cardiovascular Outcomes Trial (COLCOT) further supports residual inflammatory targeting after MI, although limited biomarker evidence and safety signals place colchicine closer to pathway-informed secondary prevention than to proof of broad inflammation suppression [244]. Metabolic therapies raise the parallel requirement that clinical benefit, tissue pathway engagement and inflammatory mediation be evaluated as separate evidence layers. The therapeutic evidence is therefore divided by what is being targeted: metabolic programs, inflammatory outputs, clinically used dual-action drugs, or patient phenotypes. These evidence layers are organized in Table 3, which separates the targeted module and disease compartment from evidence status and the corresponding therapeutic interpretation boundary.

**Table 3:** Translational and clinical evidence framework for metabolic-inflammatory therapeutic strategies in cardiovascular disease

Therapeutic strategy/agent	Primary module	Disease context/compartment	Evidence layer/status	Therapeutic implication/interpretation boundary	Key refs
SGLT2 inhibitors and ketone-related metabolic mechanisms	Cardiorenal-metabolic unloading and substrate remodeling with SGLT2 inhibition; ketone oxidation and BHB-related signaling as candidate mechanisms or adjunct metabolic strategies	HFrEF, HFmrEF and HFpEF; kidney–systemic circulation–heart axis; myocardial ketone flux remains incompletely assigned	Outcome-level HF trial evidence for SGLT2 inhibitors; metabolomic and invasive hemodynamic studies; state-of-the-art synthesis of therapeutic ketosis and small exogenous-ketone trials	SGLT2 inhibitors have robust clinical efficacy in HF, but their benefit cannot be attributed specifically to increased myocardial ketone oxidation. Ketone supplementation and therapeutic ketosis remain mechanistically plausible but are supported mainly by short-term physiological or surrogate-endpoint data; ketoacidosis risk, dietary context and patient selection remain important boundaries	[245–256]
GLP-1 receptor agonists, e.g., semaglutide	Adipometabolic inflammation, weight reduction, vascular immune modulation and systemic inflammatory-risk remodeling	Established CVD with overweight/obesity; obesity-related HFpEF; adipose–vascular–systemic inflammatory axis	Outcome-level evidence in obesity-related AS; phenotype-specific randomized evidence in obesity-related HFpEF; secondary inflammatory and mechanistic studies	Supports cardiovascular risk reduction and improvement in symptoms, functional limitation and body weight in selected obesity-related phenotypes. Whether anti-inflammatory effects directly mediate clinical benefit remains unresolved; tolerability and discontinuation require consideration	[257–262]
IL-1β/IL-6 cytokine-axis targeting	NLRP3-upstream IL-1β–IL-6–CRP cascade; IL-6 ligand/receptor signaling	Post-MI residual inflammatory risk; CKD or acute MI biomarker-enriched settings; innate immune and systemic cytokine compartments	Outcome-level evidence for IL-1β blockade; negative comparator evidence for nonspecific anti-inflammatory therapy; biomarker and substudy evidence for IL-6 inhibition	Supports pathway-specific inflammatory-risk targeting rather than broad cytokine suppression. Clinical translation is constrained by patient selection, pathway engagement, infection risk, cost and immune suppression; TNF-α blockade provides a cautionary example that cytokine elevation alone does not establish safe targetability	[242, 243, 263–271]
Colchicine	Broad innate immune and neutrophil-related inflammatory modulation; microtubule-dependent suppression of inflammatory signaling	Recent MI and chronic coronary disease; systemic innate immune and vascular inflammatory-risk compartments	Outcome-level clinical evidence and meta-analyses in secondary prevention; acute STEMI/PCI setting provides a cautionary contrast	Supports residual inflammatory-risk reduction in selected coronary phenotypes. The therapeutic signal is less pathway-specific than cytokine blockade and depends on timing, cumulative exposure, renal function, drug tolerance and gastrointestinal safety	[244, 272–277]
Direct NLRP3 inhibition, e.g., dapansutrile/OLT1177	Inflammasome activation, caspase-1 signaling, IL-1β/IL-18 maturation and pyroptotic execution	Stable HFrEF or selected inflammatory CVD states; inflammasome-active immune or cardiac compartments	Early clinical/exploratory evidence supported by mechanistic and preclinical studies	May attenuate stress-amplified inflammatory execution, but short-term safety and pharmacodynamic signals do not establish clinical efficacy. NLRP3 should be viewed as a context-dependent amplifier rather than a universal final common pathway	[28, 278–280]
PFKFB3/glycolysis modulation, e.g., PFK158, 3PO, 2-DG	Pathological glycolysis in plaque macrophages/endothelium and activated fibroblasts; glycolysis–HIF-1α–IL-1β and fibroblast glycolytic programs	Atherosclerotic plaque vulnerability and post-MI fibrosis; macrophage, endothelial and fibroblast compartments	Preclinical and mechanistic evidence	May stabilize plaques or attenuate fibroblast activation in selected injurious cell states. Because glycolysis may also support acute cardiomyocyte survival, targetability depends critically on disease stage, dose, timing and cell-type selectivity	[13, 19, 96, 138]
MPC/MCT4–lactate handling, e.g., VB124	Pyruvate–lactate routing, MCT4-dependent lactate export and lactate-linked hypertrophic remodeling	Stress-induced hypertrophic remodeling and HF models; cardiomyocyte compartment	Preclinical evidence	MCT4 inhibition may attenuate experimental hypertrophic remodeling by correcting pyruvate–lactate imbalance. This strategy is not established as clinical therapy, and stress-induced remodeling models should not be overextended to clinical or genetic HCM	[125]
FAO modulation, e.g., trimetazidine; etomoxir as a cautionary tool compound	FAO mismatch, substrate flexibility, mitochondrial capacity and tool-compound specificity	HFrEF, with HFpEF as a cautionary contrast; cardiomyocyte and immune-cell experimental contexts	Mixed clinical and preclinical evidence	Partial FAO modulation may be relevant in selected HFrEF contexts, but HFrEF findings should not be extrapolated to HFpEF. Etomoxir-based studies require caution because high-dose effects may reflect CPT-1-independent disruption of CoA homeostasis	[281–284]
Icosapent ethyl and experimental lipid-mediator modulation	Residual lipid-inflammatory risk; formulation-specific omega-3 biology; ceramide/S1P as mechanistic lipid mediators	Statin-treated patients with elevated triglycerides or residual ischemic risk; systemic lipid-inflammatory axis and plaque macrophage lipid-mediator compartment	Outcome-level evidence for selected icosapent ethyl formulation; negative comparator evidence for mixed omega-3 formulations; mechanistic evidence for ceramide/S1P signaling	Clinical benefit with icosapent ethyl should not be generalized to all omega-3 formulations or attributed solely to anti-inflammatory lipid mediators. Direct targeting of ceramide/S1P pathways remains experimentally promising but lacks comparable cardiovascular outcome evidence	[153–156, 285–287]
BCAA catabolic targeting, e.g., BCKDK inhibition/BT2	BCAA/BCKA accumulation, BCKDK–BCKDH control, mitochondrial stress and lymphatic endothelial BCAA catabolism	HFrEF/pressure-overload models; obesity/hypertension-associated HFpEF lymphatic endothelial mechanism; IRI risk context	Preclinical and compartment-specific evidence	Restoring BCAA catabolic flux may reduce stress remodeling in experimental HF and preserve lymphatic integrity in HFpEF-like disease. This remains a preclinical strategy; phenotype, compartment and disease-stage assignment are essential	[121, 137, 288]
Amino-acid redox buffering, e.g., glycine/DT-109/GSH support; homoarginine	Amino-acid-supported glutathione synthesis, macrophage redox buffering and T-cell immunometabolic modulation	Atherosclerosis and immune-redox models; macrophage and T-cell compartments	Preclinical evidence	May reduce oxidative stress, plaque inflammation or immune-cell activation in experimental models. These approaches should be framed as compartment-specific metabolic repair rather than nonspecific nutrient replacement	[141, 289]
Metformin/AMPK-centered macrophage modulation	AMPK-dependent regulation of acute macrophage inflammatory responses and AMPK–ATF1-linked atheroprotective macrophage programming	Macrophage-centered inflammatory models; normoglycaemic atherosclerosis models; metabolic-inflammatory contexts	Mechanistic cell and animal evidence, complemented by clinical trials that assessed vascular or cardiac remodeling	Supports metformin as a sensor-level bridge between metabolism and inflammation. Current evidence does not establish metformin as a dedicated cardiovascular anti-inflammatory therapy	[290–293]

*Abbreviations:* *2-DG* 2-deoxy-D-glucose, *3PO* 3-(3-pyridinyl)−1-(4-pyridinyl)−2-propen-1-one, *AMPK* AMP-activated protein kinase, *AS* atherosclerosis, *ATF1* activating transcription factor 1, *BCAA* branched-chain amino acids, *BCKA* branched-chain α-keto acids, *BCKDH* branched-chain α-keto acid dehydrogenase, *BCKDK* branched-chain α-keto acid dehydrogenase kinase, *BHB* β-hydroxybutyrate, *CKD* chronic kidney disease, *CoA* coenzyme A, *CPT-1* carnitine palmitoyltransferase 1, *CRP* C-reactive protein, *CVD* cardiovascular disease, *FAO* fatty acid oxidation, *GLP-1* glucagon-like peptide-1, *GSH* glutathione, *HCM* hypertrophic cardiomyopathy, *HF* heart failure, *HFmrEF* heart failure with mildly reduced ejection fraction, *HFpEF* heart failure with preserved ejection fraction, *HFrEF* heart failure with reduced ejection fraction, *HIF-1α* hypoxia-inducible factor 1α, *IL-1β* interleukin-1β, *IL-6* interleukin-6, *IL-18* interleukin-18, *IRI* ischemia–reperfusion injury, *MCT4* monocarboxylate transporter 4, *MI* myocardial infarction, *MPC* mitochondrial pyruvate carrier, *mTOR* mechanistic target of rapamycin, *NLRP3* NOD-like receptor family pyrin domain-containing 3, *OLT1177* dapansutrile, *PCI* percutaneous coronary intervention, *PFKFB3* 6-phosphofructo-2-kinase/fructose-2,6-bisphosphatase 3, *S1P* sphingosine-1-phosphate, *SGLT2* sodium–glucose cotransporter 2, *STEMI* ST-segment elevation myocardial infarction, *TNF-α* tumor necrosis factor-α

### Metabolism-targeted anti-inflammatory strategies

#### Glycolysis and pyruvate-lactate handling

Glycolysis-targeted therapy separates by disease stage and responding cell type. In activated macrophages, glycolytic reprogramming can support inflammatory cytokine production: LPS-induced succinate accumulation stabilizes HIF-1α and selectively promotes IL-1β expression, while 2-DG suppresses IL-1β without broadly suppressing all inflammatory outputs [96]. In cardiovascular disease models, the signal becomes compartment-specific. In AS, PFKFB3 is enriched in vulnerable plaques and mainly localizes to macrophage- and endothelial-rich areas; PFK158 reduced glycolysis in PBMCss and promoted plaque-stabilizing features in mice [13]. In post-MI fibrosis, TGF-β1 stabilizes PFKFB3 through OTUD4, thereby increasing fibroblast glycolysis and fibrotic activation; 3PO or *Pfkfb3* knockdown attenuated this response [19]. These data place glycolysis modulation in injurious immune or fibrotic cell states; timing and cell type determine whether inhibition is protective. In early post-MI myocardium, immediate high-dose 2-DG increased cardiac rupture-related mortality, whereas delayed lower-dose treatment reduced fibrosis, making timing and dose part of the therapeutic boundary [138]. Pyruvate-lactate handling is a separate target from glycolysis blockade. MPC loss and MCT4-mediated lactate export define a cardiomyocyte pyruvate-lactate program linked to hypertrophy and HF, and MCT4 inhibition with VB124 attenuated hypertrophic remodeling in experimental models [125].

#### Lipid metabolism, FAO and sphingolipid remodeling

Lipid-targeted strategies require phenotype and tool specificity as much as the direction of FAO change. Trimetazidine, a partial FAO inhibitor, has shown possible benefit in HFrEF, although this evidence rests largely on small and older studies [281]. In invasively confirmed HFpEF, trimetazidine did not improve myocardial energetic status or exercise hemodynamics [282]. This contrast restricts FAO modulation to phenotype-specific claims and prevents direct HFrEF-to-HFpEF extrapolation. FAO-inhibitor experiments also require caution. High-dose etomoxir can impair macrophage polarization through disturbed coenzyme A (CoA) homeostasis instead of clean (CPT-1)-dependent FAO inhibition, and high-concentration etomoxir experiments can introduce additional off-target effects [283, 284]. PPAR signaling introduces isoform specificity because PPARα, PPARβ/δ and PPARγ regulate lipid handling and inflammatory tone in different cellular and disease contexts [85]. Macrophage cholesterol efflux has also been targeted through plaque-responsive delivery. A pH-responsive anti-miR-33 nanotherapy increased cholesterol efflux and modified macrophage and regulatory T-cell responses, reducing lesion burden and vulnerable-plaque features in mice [294]. Clinical lipid-inflammatory modulation is similarly formulation- and phenotype-dependent. Icosapent ethyl reduced ischemic events in selected statin-treated patients with elevated triglycerides [285]. In contrast, the EPA/DHA carboxylic acid formulation tested in a high-risk population did not reduce major cardiovascular events [286]. Sphingolipid signaling extends lipid targeting beyond fuel selection. SPHK1/2-derived S1P contributes to macrophage NLRP3 priming and IL-1β secretion, but current support remains mechanism-level evidence without CVD outcome trial validation [156].

#### Amino acid metabolism and redox buffering

Amino acid-targeted strategies need flux, redox-buffering or compartment evidence, not amino-acid abundance alone. In HF, impaired BCAA catabolism and BCKA accumulation can worsen mitochondrial stress. Restoring BCAA catabolic flux through BCKDK inhibition with BT2 preserved cardiac function after pressure overload in mice, although BT2 remains a preclinical tool compound and has not been established as clinical HF therapy [288]. In IRI models, chronic BCAA/BCKA accumulation increased PPARα-dependent FAO, lipid peroxidation and injury susceptibility; this evidence identifies high-BCAA metabolic states as a possible risk context during reperfusion stress [121]. Other amino acid approaches point to immune-redox control. Glycine or DT-109 increased de novo GSH synthesis in macrophages, reduced superoxide generation and attenuated AS in *ApoE −/− *mice, supporting a glycine–GSH buffering program [289]. Homoarginine reduced atherogenesis by limiting CD4^+^ T-cell activation, migration and proliferation, placing the therapeutic signal in immune-cell behavior [141]. These data place amino acid-targeted approaches closer to compartment-specific metabolic repair than simple nutrient replacement, although clinical validation remains limited.

### Direct targeting of inflammatory pathways

#### Targeting the NLRP3 inflammasome and IL-1β axis

When metabolic correction is indirect or cell-type restricted, a second strategy is to target inflammatory outputs themselves. NLRP3 links metabolic stress to inflammatory execution. After priming and activation, it assembles with ASC and procaspase-1, leading to caspase-1 activation, IL-1β/IL-18 maturation and GSDMD-mediated pyroptosis [28, 278]. Its dominant triggers vary across CVD settings, from mtROS/mtDNA release and ionic stress in MI/IRI to cholesterol crystals, lipid stress and plaque macrophage activation in AS [28, 278]. Thus, NLRP3 is a treatment-relevant amplifier of selected stress pathways, not a final common pathway for all metabolic injury.

Direct NLRP3 inhibition is still at an early clinical stage. In stable HFrEF with mild-to-moderate symptoms, dapansutrile/OLT1177 was safe and tolerated over 14 days, with only exploratory signals in the highest-dose cohort; the study was small, single-center and not designed to test clinical efficacy [279]. At present, the stronger clinical case lies downstream, at IL-1β or related cytokine pathways; direct inflammasome inhibition remains early.

#### IL-1β blockade, colchicine and IL-6 inhibition

The clearest clinical proof for direct anti-inflammatory therapy comes from selected pathways in enriched patients. CANTOS showed that, in patients with previous MI and hsCRP ≥ 2 mg/L, canakinumab reduced vascular events without lowering LDL-C, supporting inflammatory risk as a modifiable treatment axis [263]. The implication is specific: IL-1β blockade worked in an inflammatory-risk phenotype, but its wider use is limited by patient selection, infection risk, cost and long-term immune suppression [263, 264].

Experimental studies during infarct healing have produced less uniform results. Acute IL-1β neutralization worsened subsequent LV remodeling in one MI model, whereas IL-1R1 deficiency reduced inflammatory-cell recruitment, fibrogenic signaling and chamber dilation after reperfused infarction [295, 296]. These findings differ in model and mode of pathway interruption and should not be extrapolated directly to IL-1β blockade in stable patients with residual inflammatory risk.

Colchicine provides a pragmatic example of inflammation-directed therapy with broad innate immune effects. By disrupting microtubule-dependent neutrophil activation and related innate immune signaling, it acts less selectively than cytokine blockade. Low-dose colchicine reduced cardiovascular events after recent MI [244] and in chronic coronary disease [272], giving the strongest clinical support in later secondary prevention. Meta-analyses are consistent with this pattern and suggest that treatment exposure may influence the magnitude of benefit [273, 274]. The early acute-MI setting is less convincing: colchicine started soon after percutaneous coronary intervention for ST-elevation MI did not improve cardiovascular outcomes [275]. The clinical use of colchicine is therefore shaped by timing, residual inflammatory risk, renal function and gastrointestinal tolerance.

IL-6 inhibition extends the IL-1β–IL-6–CRP cascade, but the clinical evidence is still mainly biomarker- or substudy-based. Ziltivekimab lowered systemic inflammatory and coagulation-related markers in Japanese patients with non-dialysis CKD and elevated hsCRP [265]. Tocilizumab was associated with faster neutrophil decline and weaker neutrophil-related transcriptional signals after ST-elevation myocardial infarction, with a signal toward myocardial salvage [266]. These findings support pathway engagement, but they remain short of definitive cardiovascular outcome evidence.

#### TNF-α, NF-κB/TLR4 and macrophage recruitment

Other inflammatory targets are plausible yet harder to translate. TNF-α is the clearest warning: its cardiac effects are receptor- and concentration-dependent, and anti-TNF therapy has been linked to disappointing HF data and HF adverse-event concerns in susceptible patients [267]. Pathway elevation alone is insufficient to define a safe therapeutic target.

TLR4/NF-κB and macrophage recruitment remain upstream programs and future targeting candidates, with limited status as mature systemic therapies. Macrophage-directed approaches increasingly move beyond M1/M2 polarization toward recruitment patterns, tissue niches and disease-stage-specific states, including CCR2/CCL2 and CX3CR1-related compartments [297]. A more direct proof-of-concept used fibroblast activation protein (FAP)-targeted chimeric antigen receptor (CAR) macrophages after myocardial IRI. Administered three days after injury, these cells phagocytosed activated fibroblasts, reduced myocardial fibrosis and improved cardiac function in mice [298]. These approaches require a dominant inflammatory output; they leave unresolved the metabolic stress that may continue to drive upstream signaling.

### Drugs with dual metabolic and anti-inflammatory actions

Some clinically used drugs sit between metabolic correction and direct anti-inflammatory therapy. Cytokine or inflammasome-directed approaches can suppress selected inflammatory outputs, but many patients with HF or AS also carry upstream metabolic stress. Dual-action drugs are most relevant when their effects can be linked to substrate handling, congestion, adiposity, or immune-cell regulation, with less emphasis on a single inflammatory endpoint.

#### SGLT2 inhibitors and cardio-renal-metabolic inflammation

Among agents with dual metabolic and anti-inflammatory relevance, SGLT2 inhibitors have the clearest clinical support in HF, with benefits reported across reduced, mildly reduced and preserved ejection-fraction phenotypes [245–248]. This clinical record complements metabolic studies but should not be replaced by them. The primary pharmacological target remains renal SGLT2, and circulating metabolic shifts alone cannot assign myocardial flux, tissue source or inflammatory mediation [249]. In HFrEF, dapagliflozin shifted ketone-related and short/medium-chain acylcarnitine metabolites, but biochemical ketosis was uncommon, and these changes did not consistently track with symptom improvement or natriuretic peptide reduction [250]. Across a wider EF range, dapagliflozin again altered ketone- and fatty-acid-related circulating factors, with a shared acylcarnitine signal and a weaker ketogenic pattern at higher left ventricular ejection fraction (LVEF) [251]. In invasively confirmed HFpEF, dapagliflozin lowered resting and exercise filling pressures, body weight, plasma volume and submaximal exercise lactate [252]. The available data therefore support a cardio-renal-metabolic unloading model, in which volume reduction, lower filling pressure and systemic substrate remodeling provide the main measurable links to inflammatory risk.

#### Glucagon-like peptide-1 (GLP-1) receptor agonists and adipometabolic vascular inflammation

GLP-1 receptor agonists address a more adipometabolic and vascular part of this shared metabolic-inflammatory interface. In patients with established CVD, body mass index (BMI) ≥ 27 and no diabetes, semaglutide 2.4 mg weekly reduced cardiovascular death, nonfatal MI or nonfatal stroke, although treatment discontinuation was more frequent with semaglutide [257]. In obesity-related HFpEF, the main signal was improvement in patient-reported HF symptoms, physical limitation, exercise capacity and body weight, including in patients with type 2 diabetes [258, 259]. The pooled analysis extends this pattern by showing concordant improvement in overall clinical status, body weight, walking capacity, natriuretic peptide burden and systemic inflammation [260]. Inflammatory profiling further showed that CRP declined across baseline inflammatory strata, linking semaglutide to an obesity-related inflammatory-risk phenotype while leaving direct inflammatory mediation to be tested more specifically [261]. The supportive vascular biology is consistent with this phenotype: semaglutide altered epicardial fat exosome cargo, reduced fatty acid-binding protein 4 and changed neutrophil adhesion and migration behavior [262].

#### Metformin/AMPK and other metabolic-inflammatory modulators

The evidence is less clinically direct for metformin/AMPK. Postler et al. showed that metformin dampened the acute LPS response in primary macrophages and macrophage-like cells through AMPK, independently of the HIF-1α/IL-10 mechanism described during chronic LPS exposure [290]. Seneviratne et al. placed this idea in AS, where metformin suppressed lesions in normoglycaemic *Ldlr −/− *mice through a haematopoietic AMPK–activating transcription factor 1 (ATF1), M2-like macrophage program [291]. These studies position metformin as a sensor-level bridge between metabolism and inflammation without establishing it as a cardiovascular anti-inflammatory therapy. Phenotype selection remains central, whether the target is renal-metabolic congestion in HF, obesity-related HFpEF with inflammatory enrichment, or macrophage-centered atherosclerotic biology that remains largely preclinical [252, 260, 261, 291].

### Combination therapy and phenotype-guided translation

Combination strategies should not be inferred from the number of pathways affected. Preclinical studies show that timing and cellular compartment can reverse therapeutic effects: delayed low-dose glycolysis inhibition attenuated post-MI fibroblast activation and fibrosis, whereas early high-dose 2-DG increased rupture-related mortality [138]. Nucleophosmin 1 (NPM1)-directed macrophage reprogramming and dimethyl fumarate-mediated modulation of macrophage and fibroblast oxidative metabolism provide additional repair-oriented strategies, but both remain agent-specific and lack cardiovascular outcome validation [299, 300]. Similar limitations apply to trimetazidine, etomoxir and BCAA- or glycine-related interventions, whose evidence remains phenotype-specific or predominantly mechanistic [137, 282, 284, 289].

Clinical anti-inflammatory trials illustrate a second requirement: the intervention must engage the pathogenic pathway in the selected population. IL-1β blockade reduced cardiovascular events in patients with residual inflammatory risk, whereas low-dose methotrexate did not improve outcomes in a broader cardiometabolic population [242, 243]. Colchicine efficacy also varies with clinical timing [244, 272, 275], and TNF-α blockade remains cautionary in patients susceptible to HF [267].

Combination therapy should therefore be tested in phenotypes with a predefined overlap of metabolic and inflammatory drivers rather than assembled empirically. SGLT2 inhibitors and GLP-1 receptor agonists may provide an outcome-supported clinical foundation in appropriate HF, diabetic or obesity-related phenotypes [249, 252, 257, 260, 261], but additive anti-inflammatory benefit requires dedicated trials. Such studies should demonstrate target engagement, distinguish systemic biomarker change from tissue-level mechanism and use clinical endpoints capable of establishing disease modification.

## Conclusion

Metabolism–inflammation crosstalk in cardiovascular disease is a staged, cell-specific process linking tissue stress, inflammatory signaling and metabolic programs. In the inflammatory layer, danger-signal sensing and NF-κB-related priming initiate the response; inflammasome and cytokine modules then amplify it; chemokines and adhesion molecules organize leukocyte entry; and efferocytosis or failed resolution helps determine whether the response supports repair or progresses toward persistent inflammation and remodeling [5–8, 27, 45]. Metabolic context gives this sequence its disease-specific meaning. Cardiomyocytes under ischemic or pressure stress, activated endothelial cells, plaque macrophages, T cells and fibrotic cardiac fibroblasts do not use glycolysis, lipid handling or redox buffering in the same way. The emphasis therefore stays on pathway use, measurement site and supported tissue response.

The pathways reviewed here matter because they change how cells sense stress and communicate injury. Glycolysis, lipid oxidation and amino-acid metabolism become relevant to inflammation when they alter substrate use, redox control, biosynthetic routing, metabolite signaling or cell behavior. Cellular source and timing shape their effects. A glucose-related shift may support acute cardiomyocyte survival, whereas lipid or amino-acid remodeling may amplify stress signaling when mitochondrial handling, inflammatory activation or tissue repair becomes dysregulated. The metabolic sensors and signaling metabolites discussed above therefore function as defined links between metabolic state and inflammatory or reparative output [1, 2, 20, 47].

The disease sections translate these principles into concrete programs. MI/IRI illustrates the timing problem most clearly: early glucose routing may support cardiomyocyte survival, whereas later lipid-derived injury activates different inflammatory routes [10–12]. In HF, substrate use differs by phenotype and compartment; immune-cell respiration, macrophage–cardiomyocyte IL-1β–mtROS signaling and lymphatic endothelial BCAA catabolism show why cardiomyocyte energetics alone cannot explain all HF states [137, 176, 178]. In AS, plaque metabolism combines endothelial, macrophage, VSMC and T-cell programs, so inflammatory glycolysis, defective lipid clearance, itaconate-linked restraint and systemic thrombotic metabolites may coexist without forming a single plaque phenotype [13, 129, 136, 140, 201]. For hypertrophic remodeling and fibrosis, the key distinction is that HCM patient data, stress models and fibroblast-driven fibrosis support related but non-equivalent claims [14–19, 128].

Therapeutic translation is deliberately narrower than simply “target metabolism” or “suppress inflammation.” Clinical trials reinforce this principle: benefit is strongest when pathway engagement and patient phenotype align, as illustrated by inflammatory-risk selection for IL-1β blockade [242] and the failure of nonspecific anti-inflammatory suppression to improve outcomes [243]. For metabolic interventions, the same standard applies: clinical efficacy, tissue pathway engagement and inflammatory mediation should be evaluated separately. Stable-isotope tracing, single-cell and spatial multiomics, cell-specific perturbation and phenotype-enriched trials should be used together to identify when a metabolic-inflammatory program is compensatory, when it becomes pathogenic and which patients actually carry that program. Future progress will depend on identifying the cell state, disease phase and clinical phenotype in which a given metabolic-inflammatory axis is truly pathogenic.

## Data Availability

Not applicable.
